# ADAR1‐HNRNPL‐Mediated CircCANX Decline Promotes Autophagy in Chronic Obstructive Pulmonary Disease

**DOI:** 10.1002/advs.202414211

**Published:** 2025-03-17

**Authors:** Ting‐Ting Chen, Yuan‐Yuan Wei, Jia‐Ying Kang, Da‐Wei Zhang, Jing‐Jing Ye, Xi‐Shi Sun, Mei Hong, Wen‐Ting Zhang, Hui‐Mei Wu, Zhen‐Xing Ding, Guang‐He Fei

**Affiliations:** ^1^ Department of Respiratory and Critical Care Medicine First Affiliated Hospital of Anhui Medical University Hefei Anhui Province 230022 China; ^2^ Key Laboratory of Respiratory Diseases Research and Medical Transformation of Anhui Province Hefei Anhui Province 230022 China; ^3^ Emergency Medicine Center Affiliated Hospital of Guangdong Medical University Zhanjiang Guangdong Province 524000 China; ^4^ Department of Geriatric Respiratory and Critical Care Medicine First Affiliated Hospital of Anhui Medical University Hefei Anhui Province 230022 China; ^5^ Department of Emergency Medicine First Affiliated Hospital of Anhui Medical University Hefei Anhui Province 230022 China

**Keywords:** autophagy, chronic obstructive pulmonary disease, CircCANX, CircRNA biogenesis, nonsense‐mediated mRNA decay, stress granule

## Abstract

Chronic obstructive pulmonary disease (COPD) is a characteristic chronic airway inflammatory disease that worsens over time, however, there are currently limited clinical therapeutics to suspend its progression. Circular RNAs (circRNAs), which have emerged as functional regulators in various diseases, including COPD, may server as new pharmacological targets in COPD. Here, it is identified a nuclear circRNA, circCANX, that is preferentially decreased in COPD. The linear splicing of *CANX* pre‐mRNA, enhanced by the ADAR1‐HNRNPL interaction, is responsible for the circCANX decline. Clinically, the higher circCANX expression is associated with a worse lung function index of FEV_1_/FVC among patients with COPD. CircCANX suppresses autophagy and stress granule (SG) formation to strengthen inflammation of COPD in vivo and in vitro. Mechanistically, circCANX recruits the tumor suppressor protein P53 (P53) mRNA and RNA helicase upstream frameshift 1 (UPF1) to form a ternary complex, which mediates *P53* mRNA degradation through nonsense‐mediated mRNA decay (NMD) process. Together, this study reveals an important circCANX‐mediated regulatory mechanism in COPD, and provides new insights into the potential of circRNA‐based drug and biomarker development for COPD.

## Graphical abstract

ADAR1‐HNRNPL interaction‐repressed circCANX recruits *P53* mRNA and UPF1 to assemble a complex in nuclear. This complex promotes *P53* mRNA degradation through the NMD pathway, and finally reduces autophagy and stress granules formation to further regulate inflammation. In summary, it presents an important circRNA‐induced mechanism of inflammation regulation that links circRNA biogenesis, mRNA decay, autophagy, and stress granules formation in COPD.

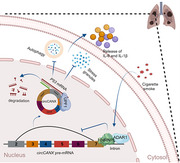

## 1 Introduction

Chronic obstructive pulmonary disease (COPD) remains the leading cause of death worldwide.^[^ ^1^, ^2^ ^]^ Inhaled particulate matter, such as cigarette smoke (CS), and genetic, developmental, and social factors contribute to the risk of COPD.^[^ ^3^ ^]^ Although the presentation of COPD is heterogeneous, prominent symptoms include persistent and often progressive airflow obstruction caused by chronically exaggerated inflammation in the lungs and abnormalities of the airways and alveoli.^[^ ^4^ ^]^ Inflammation is a pernicious factor in the COPD progression.^[^ ^5^, ^6^ ^]^ Contemporary COPD guidelines emphasize the prevention of exacerbations, which are crucial for improving poor prognosis.^[^ ^7^ ^]^ Unfortunately, there are limited clinical therapeutics for preventing pathological changes in the lungs.^[^ ^8^, ^9^ ^]^ Further research on the epigenetic mechanisms underlying COPD is urgently needed to advance pharmacological treatments.

Autophagy can be induced by cellular stress and plays multiple roles in COPD and inflammation through cellular homeostasis and survival mechanisms.^[^ ^10^, ^11^, ^12^ ^]^ During lung inflammation, autophagy activation appears to be a protective regulator of the host response to pathogens.^[^ ^13^, ^14^ ^]^ Although the tumor suppressor protein P53 (P53) is functionally linked to autophagy,^[^ ^15^, ^16^ ^]^ the underlying mechanisms remain unclear. Cellular stressors, including inflammation, regulate the formation of stress granules (SGs), which act as intracellular defense regulators to help cells recover from stress.^[^ ^17^, ^18^ ^]^ During SG assembly, stress granule assembly factor 1 (G3BP1) is the key nucleator and is abundant in the SGs.^[^ ^19^ ^]^

Cellular homeostasis involves a mRNA surveillance process called nonsense‐mediated mRNA decay (NMD).^[^ ^20^ ^]^ Thus, eukaryotic cells are protected by reducing the production of harmful proteins translated from prematurely terminated codon‐bearing transcripts.^[^ ^21^ ^]^ RNA helicase upstream frameshift 1 (UPF1) are essential NMD‐related components in all eukaryotic organisms.^[^ ^20^ ^]^

Circular RNAs (circRNAs) are unusually stable RNAs generated by the back‐splicing of host exons.^[^ ^22^ ^]^ Their production mutually regulates and competes with linear mRNA splicing.^[^ ^23^ ^]^ Depending on their localization and interactions, circRNAs have multiple functions, including transcription, splicing, and translation, and act as scaffolds and sponges.^[^ ^24^, ^25^ ^]^ Accumulating evidence has demonstrated that dysregulated circRNAs exhibit critical functions in a variety of diseases.^[^ ^26^, ^27^ ^]^ Such as circTET2 modulates the lipid metabolism to promote the proliferation of chronic lymphocytic leukemia,^[^ ^26^ ^]^ circVAMP3 contributes metastasis to inhibit hepatocellular carcinoma progression.^[^ ^27^ ^]^ However, the underlying mechanism of circRNAs and their biogenesis and regulation are still lacking in COPD.

To address this gap, here, we performed in‐depth analyses on the circRNome of patients with COPD to identify the potential roles of circRNA and associated key molecular mechanisms. We identified a novel circRNA, circCANX, was downregulated in COPD, that caused by the ADAR1‐HNRNPL interaction enhanced alternative splicing from *CANX* pre‐mRNA to mRNA. Among patients with COPD, the higher circCANX expression was associated with a worse FEV1/FVC ratio. Mechanistically, circCANX suppressed autophagy and SG accumulation to promote inflammation in vivo and in vitro. This may be due to that circCANX recruited *P53* mRNA and UPF1 to form a ternary complex, mediating *P53* mRNA degradation through NMD‐related decay. In summary, this study provides new insights into the roles of circRNA in COPD and their potential clinical value.

## 2 Results

### 2.1 CircCANX is Downregulated in CSE‐Treated Cells and Patients with COPD

**Figure 1:**
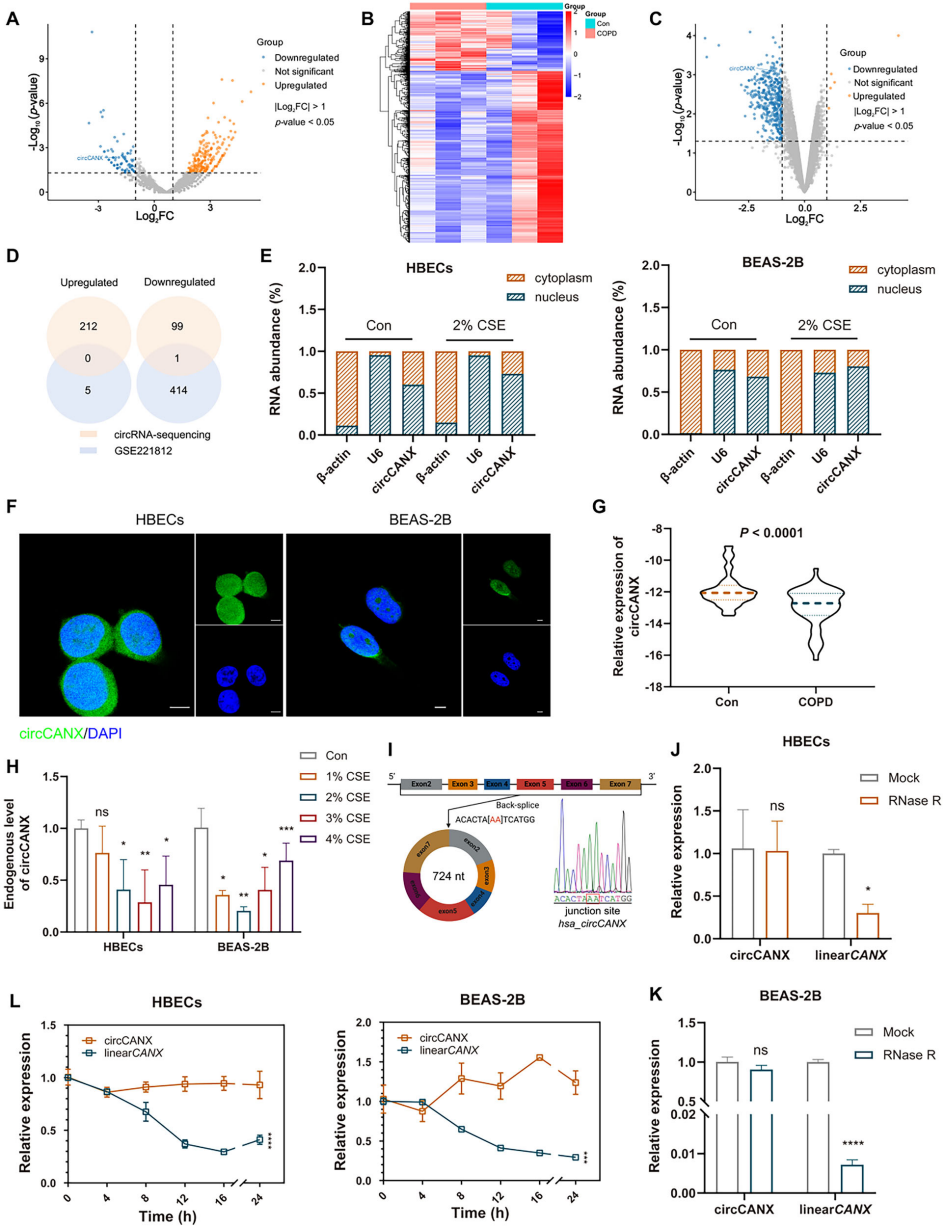
CircCANX is downregulated in CSE‐treated cells and patients with COPD. A) Volcano plots showing the differentially expressed circRNAs in patients with COPD and healthy controls. B) Heatmap of differentially expressed circRNAs between patients with COPD and healthy controls (GSE221812). C) Volcano plots showing the differentially expressed circRNAs (GSE221812). D) Venn diagram of differentially expressed circRNAs (circRNA‐sequencing and GSE221812). E) RT‐qPCR detection of circCANX in control and 2% CSE‐treated HBECs and BEAS‐2B cells. *β‐actin* mRNA and *U6* RNA were used as reference RNAs for the cytoplasm and nucleus, respectively. F) Subcellular localization of circCANX (FITC‐labeled probe) revealed by FISH in HBECs and BEAS‐2B cells. DAPI was used to stain cell nuclei. Scale bar: 5 µm. G) Relative expression of circCANX in blood samples of patients with COPD (*n* = 52) and healthy controls (*n* = 56) were detected by RT‐qPCR. H) The endogenous circCANX levels in CSE‐treated HBECs and BEAS‐2B cells were detected by RT‐qPCR. I) Schematic of back splicing of circCANX. J‐K) circCANX and linear*CANX* expression were detected in HBECs and BEAS‐2B cells after RNase R treatment. L) Relative circCANX and linear*CANX* expressions were detected after actinomycin D treatment. Data are presented as mean ± SD values. *p < 0.05, **p < 0.01, ***p < 0.001, ****p < 0.0001.

To explore functional circRNAs in COPD, we performed circRNA sequencing of blood samples from patients with COPD and healthy controls. Following the criteria of the absolute value of log_2_ (fold‐change) > 1 and *p*‐value < 0.05 between the two groups, we identified 311 differentially expressed circRNAs, including 212 upregulated and 99 downregulated circRNAs (**Figure** **1**A). We also analyzed published COPD circRNA datasets (GSE221812) with the criteria of the absolute value of log_2_ (fold‐change) >1 and *p*‐value < 0.05 and found five upregulated and 414 downregulated circRNAs (Figure 1B,C). Combining these two results, we found that circCANX (hsa_circ_0001564) was observably downregulated in patients with COPD (Figure 1D).

To evaluate the cell‐type expression of circCANX in COPD, we co‐located FITC‐labeled circCANX with markers of lymphocytes (CD3), macrophages (F4/80), and epithelial cells (CK18). The results showed that circCANX was expressed in lymphocytes, macrophages, and epithelial cells (Figure S1A–C, Supporting Information). Nuclear and Cytoplasmic separation assay revealed that circCANX was mainly localized to the nucleus of HBECs and BEAS‐2B (Figure 1E). The FISH assay results were consistent with the above results, showing that circCANX is predominantly distributed in the nucleus (Figure 1F). We further detected lower circCANX expression in the blood of patients with COPD (*n* = 52) than in normal controls (*n* = 56) using RT‐qPCR (Figure 1G), and the results were consistent with the circRNA‐sequencing data. Association analysis in patients with COPD revealed that circCANX expression correlated with the FEV1/FVC% ratio (**Table** **1**). Next, we examined the endogenous circCANX levels in HBECs and BEAS‐2B cells, the results showed downregulation in cells after cigarette smoke extract (CSE) treatment (Figure 1H).

**Table 1:** Association of circCANX expression with clinical information of patients with COPD (*n* = 52).

Clinical information	circCANX expression, Low High	*p*‐value
All cases	26, 26	
Gender		
Male	19, 22	
Female	7, 4	
Age (year)	72.04 ± 8.825, 71.69 ± 8.921	0.8887
BMI	21.22 ± 3.957, 21.14 ± 3.190	0.9370
Smoking status	16.54 ± 14.68, 18.08 ± 14.97	0.7099
FEV_1_/FVC (%)	55.14 ± 11.47, 47.12 ± 9.101	0.0074^a)^
FEV_1_ (%) predicted	40.75 ± 15.57, 36.62 ± 16.38	0.3559
FVC (%) predicted	54.78 ± 20.84, 59.84 ± 21.20	0.3898
MMEF 75/25 (%) predicted	16.02 ± 7.599, 14.15 ± 7.702	0.3826
DLCO (%) predicted	51.63 ± 22.66, 39.36 ± 20.68	0.0947
GOLD status		
I	0, 1	
II	7, 2	
III	11, 11	
IV	8, 12	

Data are shown as the means ± SD.

^a)^ *p* < 0.01.

The circBase database results indicated that circCANX was derived from exons 2 to 7 back splicing of the *CANX* pre‐mRNA. The back‐spliced junction of circCANX was validated by Sanger sequencing (Figure 1I). To confirm circCANX has circRNA characteristics, divergent and convergent primers of circCANX and *CANX* were used to detected the circular and linear forms. RNase R and actinomycin D assays indicated that circCANX was more stable than the linear*CANX* (Figure 1J–L). Together, these findings demonstrated that circCANX is a highly stable circRNA and downregulated in COPD.

### 2.2 ADAR1‐HNRNPL Interaction‐Enhanced Linear Splicing of *CANX* Pre‐mRNA is Responsible for the CircCANX Decline

**Figure 2:**
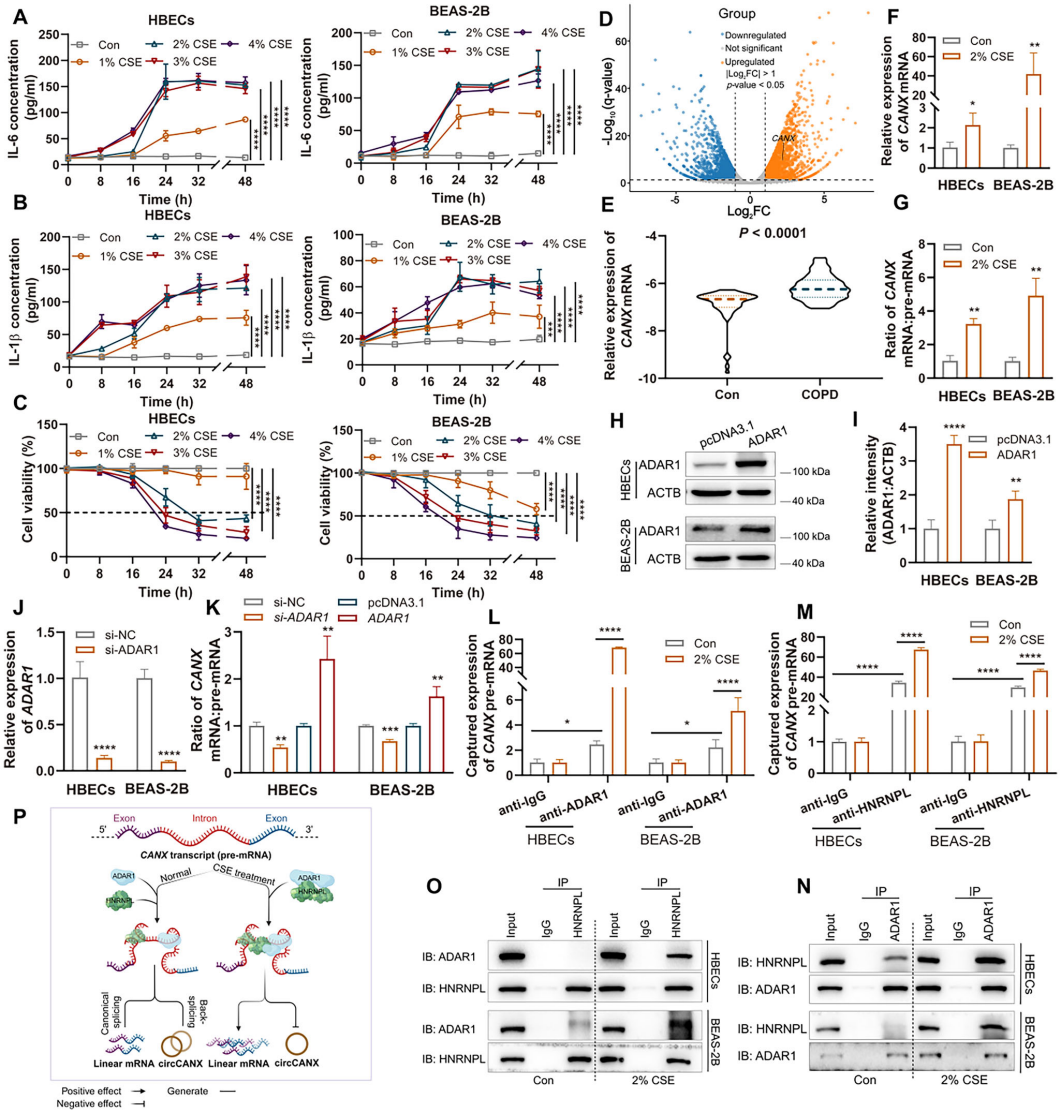
ADAR1‐HNRNPL interaction‐enhanced linear splicing of *CANX* pre‐mRNA is responsible for the circCANX decline. A,B) IL‐6 (A) and IL‐1β (B) levels were detected by ELISA assay after CSE treatment at several concentration and for different times. C) CCK‐8 assays of cell viability with the indicated treatment. D) Volcano plots showing the differentially expressed genes in blood samples of patients with COPD and healthy controls detected by RNA sequencing. E) Relative expression of *CANX* mRNA was detected by RT‐qPCR in blood samples of patients with COPD (*n* = 52) and healthy controls (*n* = 56). F) Relative expression of *CANX* mRNA was detected by RT‐qPCR in normal and 2% CSE‐treated cells. G) RT‐qPCR analysis of *CANX* mRNA to pre‐mRNA ratio in normal and 2% CSE‐treated cells. H,I) Western blot analysis and quantitation of ADAR1 after overexpressing ADAR1. J) Relative expression of *ADAR1* mRNA in cells with ADAR1 knockdown treatment. K) Ratio of *CANX* mRNA and pre‐mRNA in the indicated groups. L–M) RIP assay of *CANX* pre‐mRNA expression following ADAR1 or HNRNPL immunoprecipitation in cells with or without 2% CSE treatment. N,O) IP analysis of the ADAR1‐HNRNPL interaction in cells incubated with anti‐ADAR1 or anti‐HNRNPL and anti‐IgG. P) The schematic diagram illustrates the mechanism by which the CSE enhanced interaction of ADAR1‐HNRNPL with *CANX* pre‐mRNA, promoting canonical splicing for linear mRNA while suppressing the back‐splicing of circCANX. Data are presented as mean ± SD values. *p < 0.05, **p < 0.01, ***p < 0.001, ****p < 0.0001.

CircRNA biogenesis competes strongly with canonical linear splicing.^[^ ^23^, ^28^ ^]^ The enhancement of linear splicing can dramatically decrease the circularization efficiency of circRNAs.^[^ ^23^ ^]^ To explore the underlying mechanism of circCANX decline. We first generated CSE‐induced cells at several concentration for different times. The ELISA results revealed that the expression of IL‐6 and IL‐1β were observably increased at CSE concentration of 2%, 3%, and 4% for 24, 32, and 48 h compared with the control (**Figure** **2**A,B). Next, we used the CCK‐8 assay to evaluate cell viability and found that 3% and 4% CSE, as well as treatment times of 32 h and 48 h induced greater rates of HBECs and BEAS‐2B cells death than the 2% CSE or 24 h treatment (Figure 2C). Therefore, cells treated with 2% CSE for 24 h were used in subsequent studies.

*CANX* is generally considered a stress‐related product and overexpressed in COPD compared with healthy controls (Figure 2D).^[^ ^29^, ^30^ ^]^ Consistent results were found in the blood samples of patients with COPD and also in 2% CSE‐treated cells (Figure 2E,F). We explored the mechanism underlying circCANX downregulation in COPD and found that 2% CSE positively regulated the splicing of *CANX* pre‐mRNA, as reflected by the changes of *CANX* mRNA and pre‐mRNA ratio (Figure 2G). Previous studies revealed that the RNA‐editing enzyme ADAR1 influences the generation of certain circRNAs and regulates alternative splicing.^[^ ^31^, ^32^ ^]^ We then modulated ADAR1 levels using plasmid and siRNA to upregulate and silence the expression of ADAR1 in cells (Figure 2H–J). We confirmed that ADAR1 silencing impaired the splicing of *CANX* pre‐mRNA, whereas the opposite effect was verified upon ADAR1 overexpression (Figure 2K). RIP assays showed that ADAR1 successfully enriched *CANX* pre‐mRNA, and this enrichment was strengthened by treatment with 2% CSE (Figure 2L).

During the process of alternative splicing, heterogeneous ribonucleoprotein (hnRNP) selects the splice site and regulates alternative splicing.^[^ ^33^ ^]^ Since HNRNPL activates splicing,^[^ ^33^, ^34^ ^]^ we hypothesized that HNRNPL participates in the process of stimulative splicing in our study. Notably, HNRNPL interacted with *CANX* pre‐mRNA, which was enhanced after 2% CSE treatment (Figure 2M). We further found that ADAR1 was bound to HNRNPL after ADAR1 immunoprecipitation and the binding was enhanced in 2% of CSE cells (Figure 2N). Similarly, strengthened binding of ADAR1‐HNRNPL induced by cells treated with 2% CSE was also observed during HNRNPL immunoprecipitation (Figure 2O). Together, these results suggested that 2% CSE enhances the ADAR1‐HNRNPL interaction to promote alternative splicing from *CANX* pre‐mRNA to *CANX* mRNA, leading to a decrease in circCANX expression (Figure 2P).

### 2.3 Depletion of ADAR1/HNRNPL Inhibits Autophagy and SG Formation

**Figure 3:**
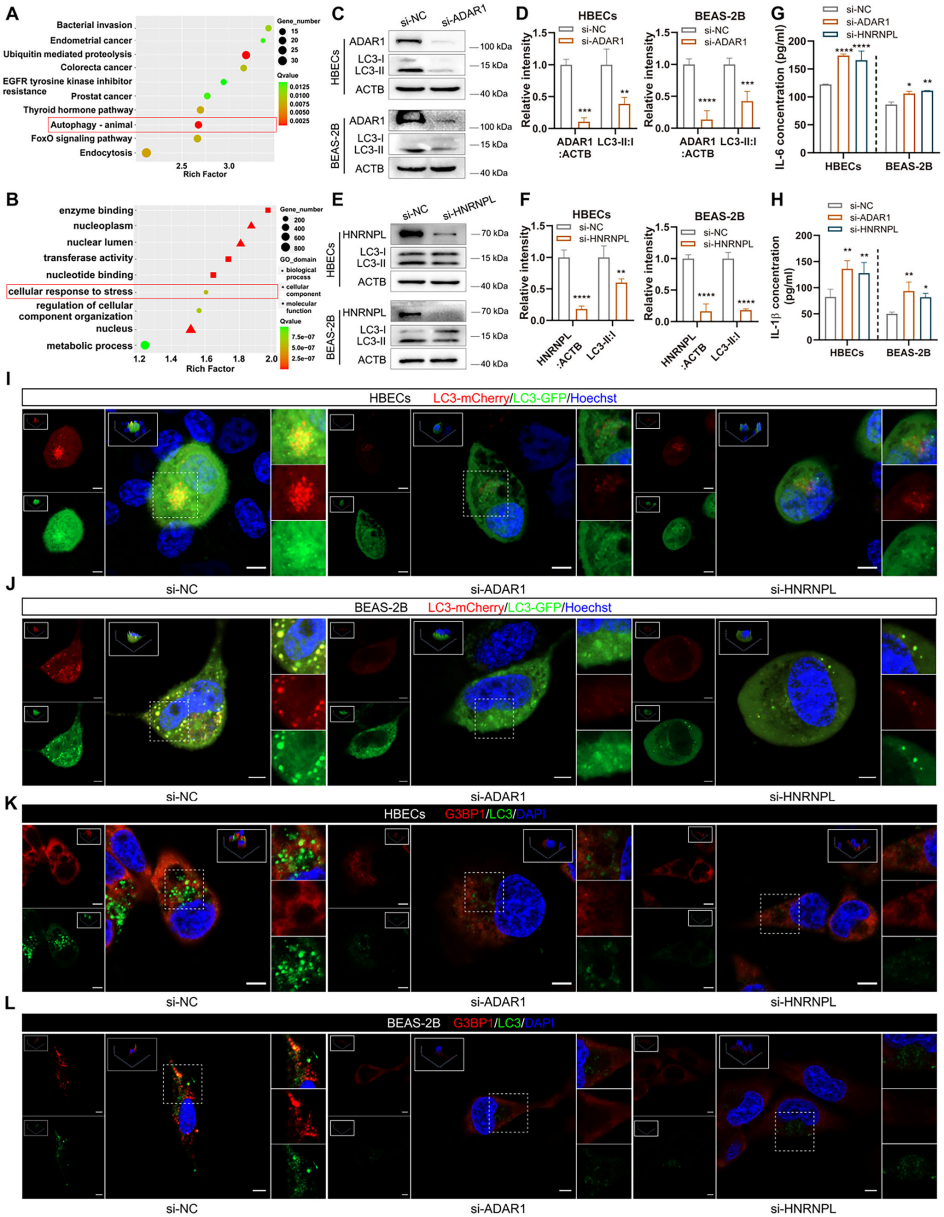
Depletion of ADAR1/HNRNPL inhibits autophagy and SG formation. A,B) Functional enrichment analysis of circRNAs‐related. C,D) Western blot analysis and quantitation of ADAR1 and autophagy‐related protein LC3 in cells with ADAR1 knockdown. E,F) Western blot analysis and quantitation of HNRNPL and LC3 after HNRNPL downregulation. G‐H) ELISAs of IL‐6 (G) and IL‐1β (H) levels after transfected with si‐ADAR1 and si‐HNRNPL. I,J) Representative images of LC3 dots after mCherry‐GFP‐LC3 transfection in the indicated groups. Hoechst dye was used to stain cell nuclei. Scale bar: 5 µm. K,L) Representative fluorescent images of G3BP1 (red) and LC3 (green) in the indicated groups. DAPI was used to stain the cell nuclei. Scale bar: 5 µm. Data are presented as mean ± SD values. *p < 0.05, **p < 0.01, ***p < 0.001, ****p < 0.0001.

Functional analysis showed high enrichment for autophagy and stress response (**Figure** **3**A,B), therefore, we first investigated the effects of CSE on autophagy. Western blotting showed that 2%, 3%, and 4% CSE treatments increased the abundance of LC3 (Figure S2A,B, Supporting Information). Similar trends were observed after transfecting mCherry‐GFP‐LC3 adenovirus to monitor autophagy flux in cells; 2%, 3%, and 4% CSE treatments increased LC3 abundance (Figure S2C, Supporting Information).

Given that the ADAR1‐HNRNPL interaction regulated the RNA competition event between *CANX* mRNA and circCANX, we next explored the role that ADAR1 and HNRNPL play in the progression of COPD. After reducing ADAR1 levels in cells using siRNA, western blot analysis showed that LC3 abundance was decreased (Figure 3C,D). Consistent results were observed in the si‐HNRNPL group; HNRNPL silencing also reduced LC3 abundance (Figure 3E,F). We then transfected cells with mCherry‐GFP‐LC3 adenovirus to monitor autophagy flux, and the results demonstrated that knockdown of ADAR1 and HNRNPL inhibited autophagy (Figure 3I,J). In addition, immunofluorescence staining results showed that the expression of G3BP1 and LC3 was reduced by the knockdown of ADAR1 and HNRNPL (Figure 3K,L). Furthermore, ELISA assay results showed that the levels of inflammatory factors IL‐6 and IL‐1β were increased by si‐ADAR1 and si‐HNRNPL transfection (Figure 3G,H). Together, these results indicated that depletion of ADAR1 and HNRNPL suppressed autophagy and SG formation and enhanced inflammation.

### 2.4 CircCANX Suppresses Autophagy and SG Formation to Promote Inflammation in CSE‐Treated Cells

**Figure 4:**
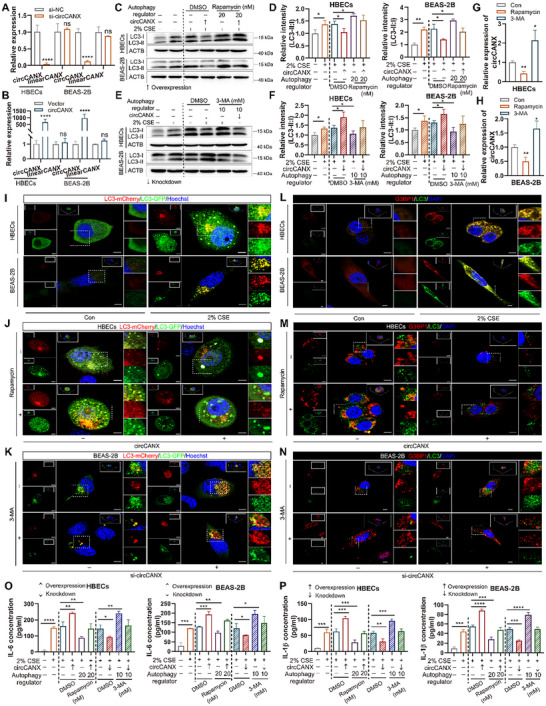
CircCANX suppresses autophagy and SG formation to promote inflammation in CSE‐treated cells. A,B) Relative expression of circCANX and linear*CANX* in HBECs and BEAS‐2B cells after circCANX knockdown and overexpression. C,D) Western blot analysis and quantitation of autophagy‐related protein LC3 in cells with circCANX overexpression and CSE and rapamycin treatment. E,F) Western blot analysis and quantitation of LC3 protein with circCANX knockdown and CSE and 3‐MA treatment. G,H) Relative expression of circCANX after rapamycin and 3‐MA treatment. I–K) LC3 dots in mCherry‐GFP‐LC3‐labeled cells in the indicated groups. Hoechst dye was used to stain cell nuclei. Scale bar: 5 µm. L–N) Immunofluorescence staining of G3BP1 (red) and LC3 (green) in the indicated groups. DAPI was used to stain cell nuclei. Scale bar: 5 µm. O‐P) ELISAs of IL‐6 (O) and IL‐1β (P) levels in cells with indicated treatments. Data are presented as mean ± SD values. *p < 0.05, **p < 0.01, ***p < 0.001, ****p < 0.0001.

To excavate the effects of circCANX on COPD, we first constructed siRNA targeting the back‐spliced sites of circCANX and an overexpression plasmid to modulate the circCANX levels in HBECs and BEAS‐2B cells. Downregulation and overexpression of circCANX were verified in cells after transfecting with the respective constructs and showing no influence on the expression of linear*CANX* (**Figure** **4**A,B).

Functional enrichment analysis demonstrated that the circRNAs were highly correlated with autophagy and stress response (Figure 3A,B). To investigate the function of circCANX in autophagy, the autophagy activator rapamycin and inhibitor 3‐MA were used in subsequent experiments. Western blot analysis results indicated that LC3 expression was negatively regulated by circCANX (Figure 4C–F; Figure S3A, Supporting Information), and 2% CSE and rapamycin treatment increased LC3 expression, which was partly rescued by circCANX overexpression (Figure 4C,D). In contrast, circCANX knockdown enhanced the promoting effect of 2% CSE and restored the inhibitory effect of LC3 downregulation via 3‐MA treatment (Figure 4E,F). We further transfected cells with mCherry‐GFP‐LC3 adenovirus, and the results demonstrated that LC3 dots increased after 2% CSE treatment (Figure 4I), and circCANX overexpression inhibited the effects on LC3 dots and partially attenuated rapamycin‐mediated promotion of autophagic flux (Figure 4J; Figure S3B, Supporting Information). In addition, circCANX knockdown increased the LC3 dots and rescued the autophagic flux inhibition caused by 3‐MA (Figure 4K; Figure S3B, Supporting Information). Similar trends were observed in immunofluorescence staining, and the expression of G3BP1 and LC3 was strengthened by 2% CSE and rapamycin treatment; such promotional effects were suspended by circCANX overexpression (Figure 4L,M). The 3‐MA‐induced inhibitory effects on LC3 expression were reversed by circCANX knockdown (Figure 4N). Notably, treatment with 3‐MA increased LC3 expression but failed to increase G3BP1 levels (Figure 4N). We next investigated whether autophagy modulates circCANX. The results indeed indicated that rapamycin reduced circCANX levels, while 3‐MA increased circCANX levels (Figure 4G,H). Furthermore, we detected the levels of inflammatory factors IL‐6 and IL‐1β, the results showed that the IL‐6 and IL‐1β levels were positively regulated by circCANX, with increased expression in cells transfected with circCANX overexpression plasmid, while the opposite results were observed in cells transfected with the si‐circCANX (Figure 4O,P; Figure S3C–E, Supporting Information), and these effects on IL‐6 and IL‐1β levels were reversed by rapamycin and 3‐MA, respectively (Figure 4O,P; Figure S3D,E, Supporting Information). Together, these in vitro results indicated that circCANX functions in autophagy, SG formation, and the inflammatory factor regulation in cells stimulated by CSE.

### 2.5 P53 is Suppressed by CircCANX and Functionally Relevant

**Figure 5:**
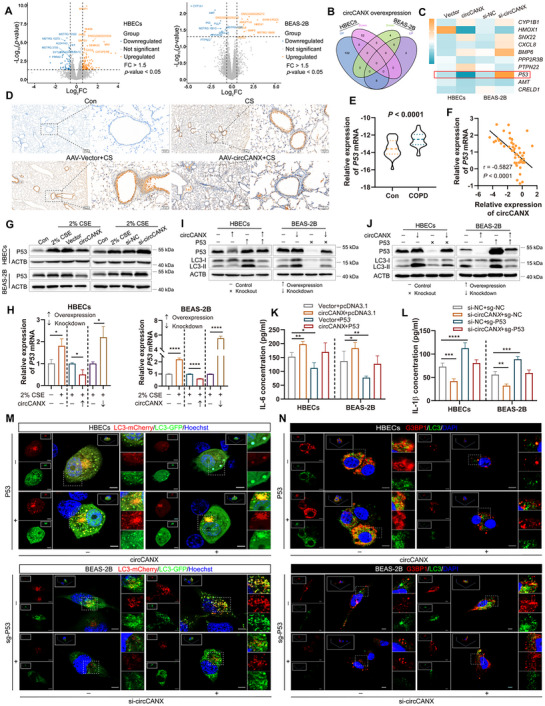
P53 is suppressed by circCANX and functionally relevant. A) Volcano plots showing the differentially expressed genes after circCANX overexpression in HBECs and BEAS‐2B cells. The top nine genes are shown. B) Venn diagram showing the overlapping number of differentially expressed genes between the two circCANX overexpression cells lines. C) The top five overlapping genes in the downregulated and upregulated groups were detected by RT‐qPCR and are shown as a heatmap after overexpressing or silencing circCANX. D) Representative immunohistochemical images of P53 (brown) in mouse lung tissues from the indicated groups. E) RT‐qPCR detection of *P53* expression in blood samples of patients with COPD (*n* = 52) and healthy controls (*n* = 56). F) Correlation between circCANX and *P53* levels in blood samples of patients with COPD. G,H) Relative expression of P53 at both the protein and mRNA levels was detected by western blotting and RT‐qPCR. I,J) Western blot analysis of P53 and LC3 after circCANX upregulation or downregulation in cells transfected with P53 plasmid or sgRNA. K,L) IL‐6 (K) and IL‐1β (L) levels were detected by ELISA assay in the indicated groups. M) Representative images of LC3 dots after mCherry‐GFP‐LC3 transfection. Hoechst dye was used to stain cell nuclei. Scale bar: 5 µm. N) Representative fluorescent images of G3BP1 (red) and LC3 (green) in the indicated groups. DAPI was used to stain the cell nuclei. Scale bar: 5 µm. Data are presented as mean ± SD values. *p < 0.05, **p < 0.01, ***p < 0.001, ****p < 0.0001.

To explore the mechanism of circCANX‐mediated autophagy and SG formation, we constructed cell lines with circCANX overexpression to perform transcriptome analysis (**Figure** **5**A). In total, eight genes were upregulated, and five genes were downregulated in both cell lines (Figure 5B). We then validated the top five candidates in both the upregulated and downregulated groups using circCANX‐overexpression and circCANX‐knockdown HBECs and BEAS‐2B cells using RT‐qPCR. Among them, only P53 appeared an accordant change which was consistent with the transcriptome analysis showed as upregulated in the circCANX‐knockdown cells but downregulated in the circCANX‐overexpression cells (Figure 5C). We also validated the expression of P53 which was perfectly negatively correlated with circCANX in the CS‐exposed mice (Figure 5D; Figure S4A,B, Supporting Information) and patient blood samples (Figure 5E,F). Next, circCANX‐suppressed P53 expression was verified by western blotting and RT‐qPCR in multiple cell lines. The results showed that 2% CSE reduced the expression of P53 at the mRNA and protein levels and upregulated circCANX restrained the P53 levels, while circCANX silencing enhanced P53 levels (Figure 5G,H; Figure S4C–E, Supporting Information).

P53 is involved in autophagy and inflammation.^[^ ^16^ ^]^ To confirm that P53 is indispensable for circCANX‐regulated functions, we performed rescue experiments. The results revealed that ectopic P53 expression impeded the inhibitory effects of P53 levels caused by circCANX overexpression, while exhausted P53 expression relieved the promotional effects induced by circCANX knockdown (Figure 5I,J; Figure S4F–H, Supporting Information). In addition, ectopic P53 expression strengthened LC3 protein, LC3 dots, and G3BP1 expression and completely abrogated the effects of circCANX, whereas the opposite effects were observed in the P53‐silenced group (Figure 5I,J,M,N; Figure S4F,G,I, Supporting Information). circCANX‐mediated IL‐6 and IL‐1β levels could be reversed by P53 manipulation (Figure 5K,L; Figure S4J–M, Supporting Information). Together, these results suggested that the circCANX inhibits autophagy and SG accumulation, and promotes IL‐6 and IL‐1β expression in multiple cell lines by suppressing its downstream effector P53.

### 2.6 CircCANX Promotes mRNA Decay by Physically Connecting the *P53* Transcript in the NMD‐Related Manner

**Figure 6:**
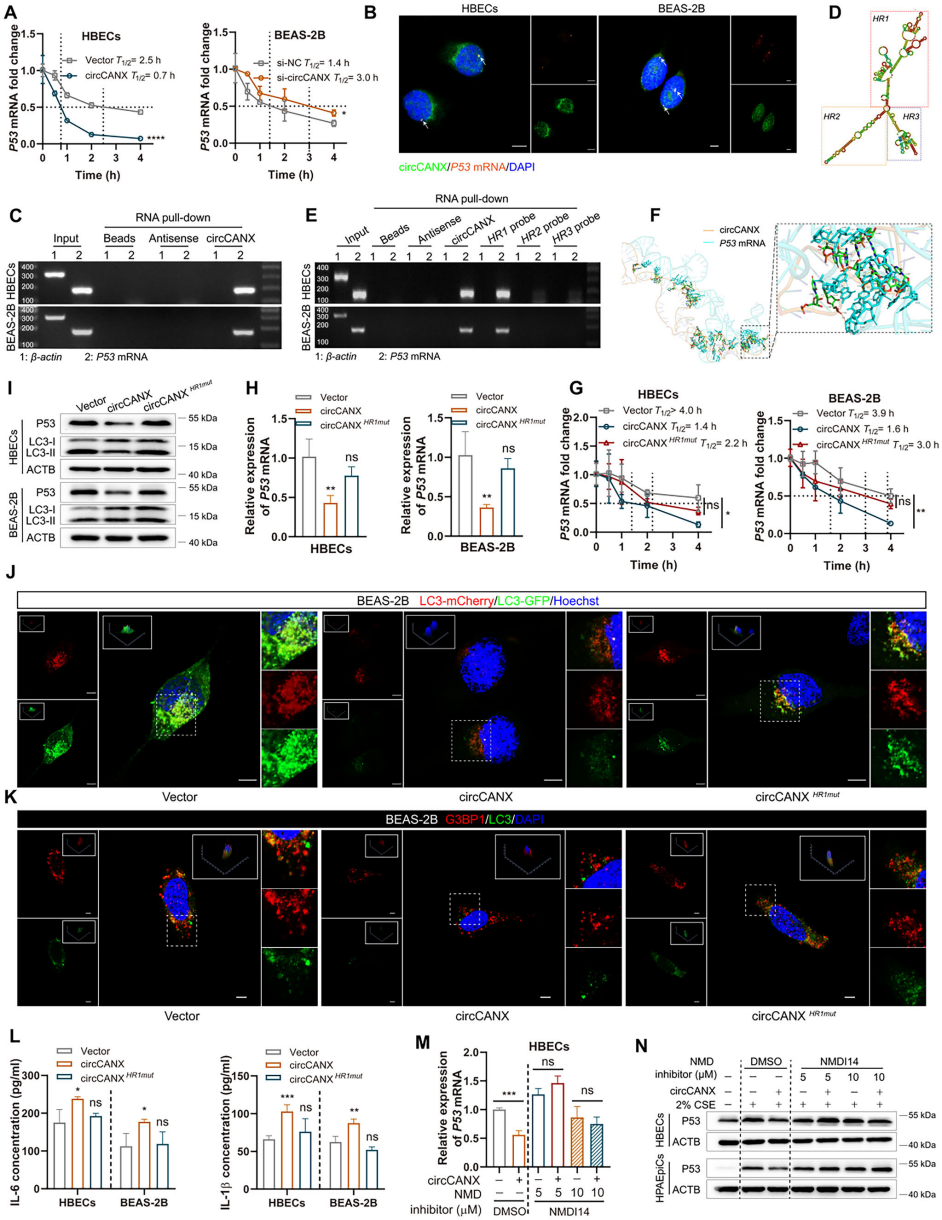
CircCANX promotes mRNA decay by physically connecting the *P53* transcript in the NMD‐related manner. A) Relative expression of *P53* mRNA at specific time in circCANX overexpression or knockdown cells treated with actinomycin D. The dashed line demonstrates the half‐life of *P53* mRNA. B) FISH images showing the co‐localization of circCANX (FITC‐labeled probe) and *P53* transcript (orange). Scale bar: 5 µm. C) RNA pull‐down assay showing the interaction between circCANX and *P53* mRNA using the circCANX and control probes. D) Predicted secondary structure and HRs (1–3) of circCANX. E) RNA pull‐down assay showing the interaction of the indicated probes and *P53* mRNA. F) Simulated interaction between circCANX and *P53* transcript. G,H) RT‐qPCR detection of *P53* mRNA in three groups (Vector, circCANX, and circCANX*^HR1mut^*) with or without actinomycin D treatment. I) Western blot analysis of P53 and LC3 in cells transfected with the indicated plasmids. J) LC3 dots in the indicated groups after transfection with mCherry‐GFP‐LC3. Hoechst dye was used to stain cell nuclei. Scale bar: 5 µm. K) Immunofluorescence staining of G3BP1 (red) and LC3 (green) in the Vector, circCANX, and circCANX*^HR1mut^* groups. DAPI was used to stain cell nuclei. Scale bar: 5 µm. L) ELISAs of IL‐6 and IL‐1β levels after transfection in the indicated groups. M,N) P53 expression in cells overexpressing circCANX or its control and treated with NMDI14 based on RT‐qPCR and western blotting. Data are presented as mean ± SD values. *p < 0.05, **p < 0.01, ***p < 0.001, ****p < 0.0001.

Considering that both P53 mRNA and protein levels were inhibited by circCANX (Figure 5G,H; Figure S4C, Supporting Information), we hypothesized that circCANX induces the transcriptional or post‐transcriptional regulation of P53. Our pathway enrichment analysis showed that circCANX‐interacting genes are highly correlated with the mRNA surveillance pathway (Figure S5A, Supporting Information). This implies that post‐transcriptional regulation is more likely.^[^ ^35^ ^]^ The actinomycin D assay confirmed that circCANX indeed promoted *P53* mRNA degradation, whereas circCANX knockdown had the opposite effect (**Figure** **6**A; Figure S5B, Supporting Information). A study indicates that circRNAs may function as molecular scaffolds to influence substrate stability.^[^ ^36^ ^]^ To elucidate the mechanism through which circCANX regulates *P53* degradation, we determined the subcellular localization of circCANX and *P53* mRNA using FISH. The results revealed marked co‐localization of circCANX and *P53* mRNA (Figure 6B). Next, we tested the possible physical interactions between circCANX and *P53* transcript using a biotin‐labeled circCANX probe. The results demonstrated that circCANX could combine with *P53* mRNA (Figure 6C).

The RNA loop is the important structure mediating the RNA interaction.^[^ ^37^ ^]^ Thus, we predicted the lowest free energy model of circCANX, constructed three hairpin (HR) probes, and matched the potential binding sites with *P53* mRNA (Figure 6D; Figure S5C, D, Supporting Information). The results revealed that HR1 was related to the circCANX*‐P53* mRNA combination, whereas HR2 and HR3 were scarcely bound to the *P53* mRNA (Figure 6E). Interaction simulations revealed a physical connection between circCANX and *P53* mRNA (Figure 6F). We constructed a circCANX plasmid containing an HR1 mutation (circCANX*^HR1mut^*). circCANX*^HR1mut^* completely lost the prodegradative effect of *P53* induced by circCANX (Figure 6G–I; Figure S5E, Supporting Information). Foreseeably, circCANX*^HR1mut^* also discarded the effects of decreasing the LC3 protein, LC3 dots, and G3BP1 expression (Figure 6I–K; Figure S5E, Supporting Information), and lost the ability of promoting the IL‐6 and IL‐1β levels (Figure 6L; Figure S5F, Supporting Information). Previous studies revealed that NMD is a vital mRNA surveillance mechanism.^[^ ^38^, ^39^ ^]^ We found that blocking the NMD pathway using the specific inhibitor NMDI14 impeded circCANX‐induced P53 downregulation (Figure 6M,N; Figure S5G,H, Supporting Information). In general, these results indicated that circCANX physically binds to the *P53* transcript and facilitates its degradation in an NMD‐dependent manner.

### 2.7 UPF1 Stabilizes the CircCANX‐*P53* mRNA Interaction by Forming a Ternary Complex

**Figure 7:**
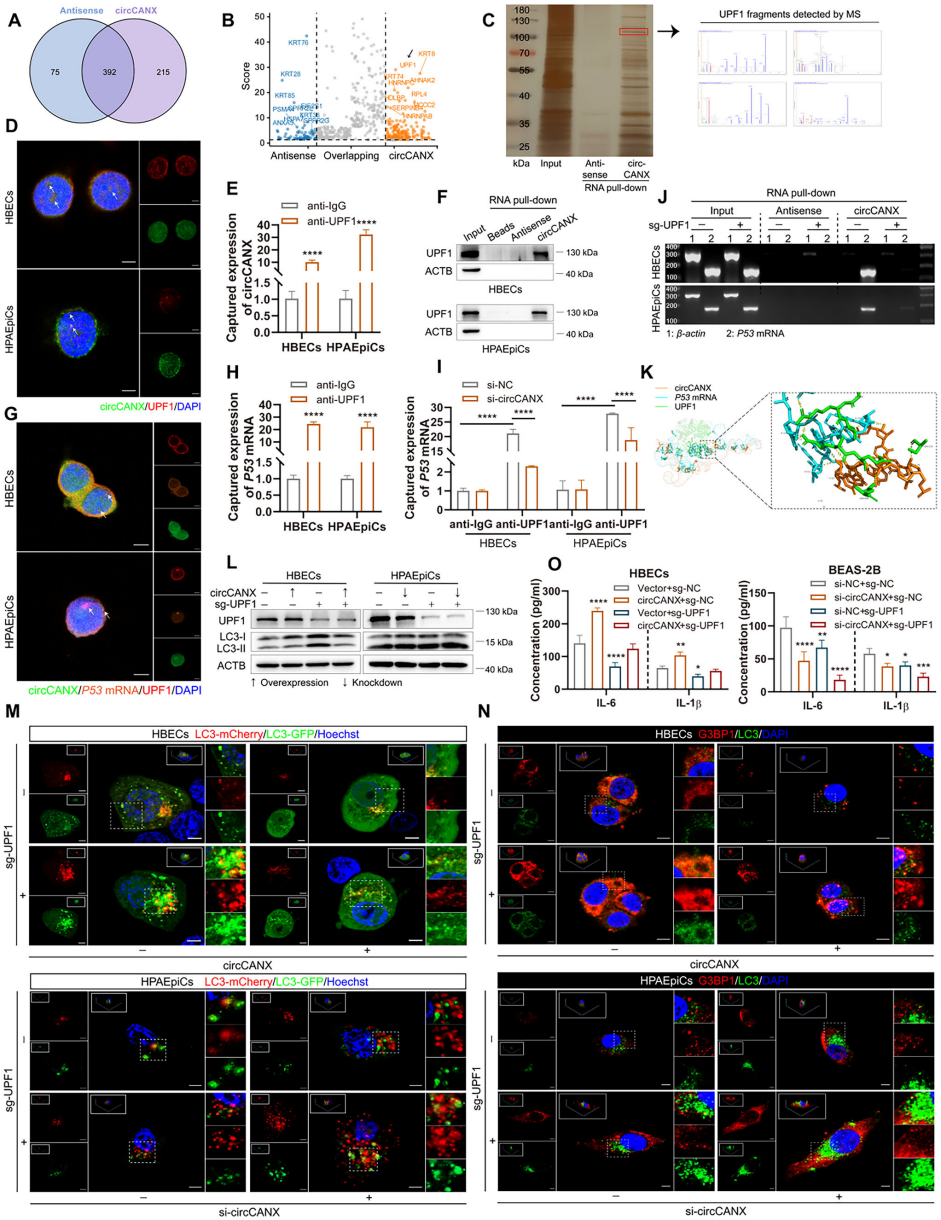
UPF1 stabilizes the circCANX‐*P53* mRNA interaction by forming a ternary complex. A) Venn diagram of differentially expressed proteins after pull‐down of circCANX and its control in HBECs using MS. B) Volcano plots showing the differentially expressed proteins and the top 10 are marked. C) circCANX‐binding proteins purified by circCANX pull‐down and visualized by silver staining. Several UPF1 peptide fragments were identified by MS. D) Co‐localization of circCANX FISH (FITC‐labeled) and anti‐UPF1 immunofluorescence (red) in the nucleus. DAPI was used to stain cell nuclei. Scale bar: 5 µm. E) RIP assay of circCANX expression after UPF1 immunoprecipitation in HBECs and HPAEpiCs. F) Expression of UPF1 after RNA pull‐down using a circCANX probe and its control. G) Co‐localization of circCANX (FITC‐labeled), *P53* transcript (orange), and UPF1 (red) in the nucleus. DAPI was used to stain cell nuclei. Scale bar: 5 µm. H,I) RIP assays using the anti‐UPF1 with or without circCANX siRNA and its control transfection. J) Interaction of circCANX and *P53* mRNA was evaluated by RNA pull‐down after the knockout of UPF1 in HBECs and HPAEpiCs cells. K) Simulated interaction between circCANX, *P53* mRNA, and UPF1. L) UPF1 and LC3 expression in cells with or without UPF1 knockout and co‐transfected with the circCANX plasmid or siRNA detected by western blotting. M) LC3 dots in mCherry‐GFP‐LC3‐transfected cells in the indicated groups. Hoechst dye was used to stain cell nuclei. Scale bar: 5 µm. N) Immunofluorescence images of G3BP1 (red) and LC3 (green) in cells transfected with the indicated plasmids or siRNA and controls. DAPI was used to stain cell nuclei. Scale bar: 5 µm. O) ELISAs of IL‐6 and IL‐1β levels after the indicated treatments. Data are presented as mean ± SD values. *p < 0.05, **p < 0.01, ***p < 0.001, ****p < 0.0001.

Since circCANX functions as a complex scaffold that directly binds to *P53* mRNA and promotes its degradation via the NMD process,^[^ ^22^ ^]^ we hypothesized that other functional cofactors may be involved. MS analysis of the circCANX interactome identified 215 candidate proteins by following the criteria of the absolute value of log_2_ (fold‐change) >1 and *p*‐value < 0.05 (**Figure** **7**A). Among these, UPF1 was ranked as the top circCANX‐binding protein and has been implicated in mRNA decay and NMD (Figure 7B; Figure S6A, Supporting Information).^[^ ^40^ ^]^ Silver staining confirmed that circCANX interacted with UPF1 (Figure 7C). Consistent results were observed in co‐localization staining, RIP, and RNA pull‐down assays (Figure 7D–F; Figure S6B, Supporting Information). To elucidate the interaction mechanism, we co‐located circCANX, *P53* mRNA, and UPF1, and the results showed overlapping images in the HBECs and HPAEpiC nuclei (Figure 7G). The RIP assay revealed an observable interaction between UPF1 and *P53* mRNA (Figure 7H; Figure S6C, Supporting Information). In addition, UPF1 immunoprecipitation successfully enriched *P53* mRNA, which was abrogated by circCANX knockdown (Figure 7I; Figure S6D, Supporting Information). After transfecting a sgRNA to knockout UPF1 levels in cells, we found that the circCANX‐*P53* mRNA interaction was completely suspended (Figure 7J). An interaction simulation was generated to demonstrate the physical connections between circCANX, *P53* mRNA, and UPF1 (Figure 7K). Finally, the deletion of UPF1 completely abrogated the effects of circCANX on autophagy, SG formation, and inflammatory factor regulation and enhanced these effects in cells with circCANX downregulation (Figure 7L–O; Figure S6E–H, Supporting Information). Together, these results demonstrated that UPF1 functions as a key regulator stabilizing the circCANX‐*P53* mRNA interaction and regulates circCANX‐mediated biological effects in cells.

### 2.8 UPF1 is Indispensable for the CircCANX‐Induced *P53* mRNA Degradation

**Figure 8:**
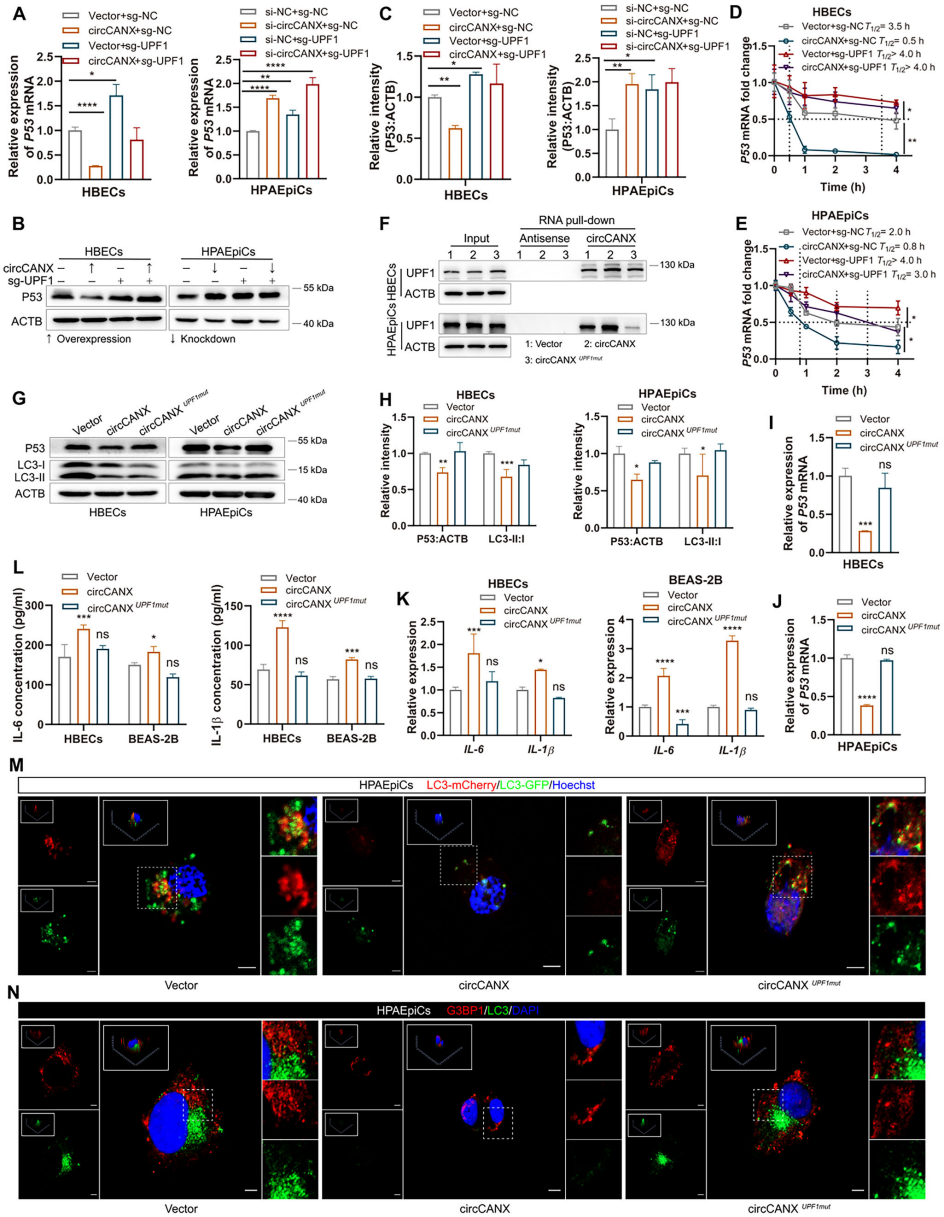
UPF1 is indispensable for the circCANX‐induced *P53* mRNA degradation. A) RT‐qPCR of the *P53* mRNA expression in HBECs and HPAEpiCs cells with or without UPF1 knockout and co‐transfection with the circCANX plasmid or siRNA. B,C) Western blot analysis and quantitation of P53 levels in the indicated groups. D,E) Relative *P53* mRNA expression in HBECs and HPAEpiCs cells after co‐transfection with the circCANX plasmid and UPF1 sgRNA or its controls. Actinomycin D was treated for the indicated times and the dashed line demonstrates the half‐life of *P53* mRNA. F) RNA pull‐down assay using the circCANX and its control probes shows enrichment of UPF1 expression in cells following the indicated treatments. G,H) Western blot analysis and quantitation of P53 and LC3 levels in the Vector, circCANX, and circCANX*^UPF1mut^* groups. I,J) RT‐qPCR of *P53* mRNA expression in the same three groups. K,L) IL‐6 and IL‐1β levels were detected by RT‐qPCR (K) and ELISAs (L) in cells transfected as indicated. M) LC3 dots in HPAEpiCs cells after mCherry‐GFP‐LC3 transfection in the indicated groups. Hoechst dye was used to stain cell nuclei. Scale bar: 5 µm. N) Immunofluorescence images of G3BP1 (red) and LC3 (green) in HPAEpiCs cells transfected as indicated. DAPI was used to stain cell nuclei. Scale bar: 5 µm. Data are presented as mean ± SD values. *p < 0.05, **p < 0.01, ***p < 0.001, ****p < 0.0001.

To further explore the role of UPF1 in the circCANX‐mediated mechanisms, we designed a series of experiments. First, UPF1 knockout completely impeded circCANX‐induced P53 downregulation at both the mRNA and protein levels, whereas the opposite trend was observed in circCANX‐knockdown cells (**Figure** **8**A–E). We then constructed a circCNAX mutation plasmid in the predicted binding sites for circCANX and UPF1 (circCANX*^UPF1mut^*). The RNA pull‐down results demonstrated that circCANX*^UPF1mut^* impaired the binding of circCANX with UPF1 (Figure 8F). Additionally, circCANX*^UPF1mut^* eliminated the effect of circCANX on *P53* mRNA degradation (Figure 8G–J). circCANX*^UPF1mut^* also lost the circCANX functions on autophagy, SG formation, and the IL‐6 and IL‐1β regulation, with shown to promote LC3 protein, LC3 dots, and G3BP1 expression (Figure 8G,H,M,N), and inhibit the IL‐6 and IL‐1β levels (Figure 8K,L). Together, these results demonstrated that UPF1 is necessary for circCANX‐mediated *P53* mRNA degradation and biological functions. We believe that circCANX directly interacts with *P53* mRNA and UPF1 to form a functional complex that regulates *P53* transcript decay.

### 2.9 CircCANX Promotes Destruction of Lung Structure and Inflammation in a Mouse Model of CS Exposure

To explore the functions of circCANX in vivo, we first established a mouse model of CS exposure by exposing mice to CS for 6 months (Figure S7A, Supporting Information). The weights of the mice were recorded monthly, and lower weights were observed in the CS group (Figure S7B, Supporting Information). Lung function detection revealed decreased values of forced expiratory volume in 20 s (FEV_20_)/forced vital capacity (FVC), FEV_50_/FVC, VC, maximal mid‐expiratory flow (MMEF), peak inspiratory flow (PIF), and inspiratory time (Ti), and increased values of functional residual capacity (FRC) and expiratory time (Te) in the CS group compared with those in the control group (Figure S7C–F, Supporting Information). Cell classification and counting in bronchoalveolar lavage fluid (BALF) showed that the number of neutrophils, macrophages, and lymphocytes increased in the CS group (Figure S7G, Supporting Information). In addition, CS exposure for 6 months induced emphysematous changes in the lungs and airway wall thickening in mice, as observed by H&E staining (Figure S7H, Supporting Information). PAS staining of the airway tissue indicated increased goblet cell proliferation in CS‐treated mice but not in the normal group (Figure S7H, Supporting Information). Masson staining revealed a high fibrosis level around the bronchi and in the interstitial regions of mouse lungs in the CS group (Figure S7H, Supporting Information). These results demonstrated that a mouse model of CS exposure was successfully constructed.

**Figure 9:**
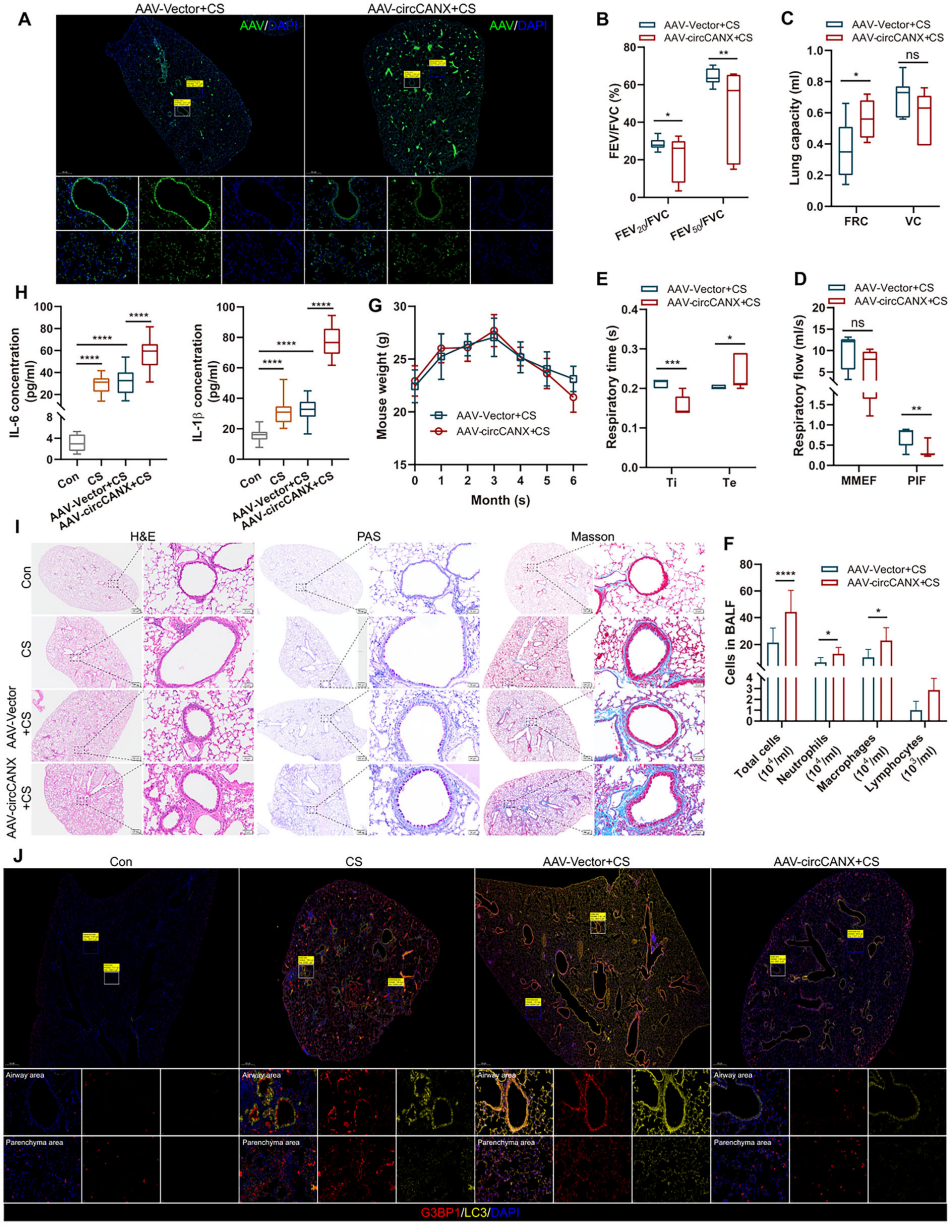
CircCANX promotes destruction of lung structure and inflammation in a mouse model of CS exposure. A) The AAV (FITC‐labeled) infection efficiency of mouse lung tissues shown in immunofluorescence images. B–E) The indicated lung function indices of the two groups (*n* = 7 mice/group). F) Cell classification and counting in BALF. G) Mouse weight was recorded every month. H) ELISAs of IL‐6 and IL‐1β levels in control (Con), CS, AAV‐vector+CS, and AAV‐circCANX+CS groups. I) Representative images of H&E, PAS, and Masson staining of mouse lungs in the indicated groups. J) Representative immunofluorescence images of G3BP1 (red) and LC3 (yellow) in mouse lungs in the four groups. DAPI was used to stain cell nuclei. Data are presented as mean ± SD values. *p < 0.05, **p < 0.01, ***p < 0.001, ****p < 0.0001.

**Figure 10:**
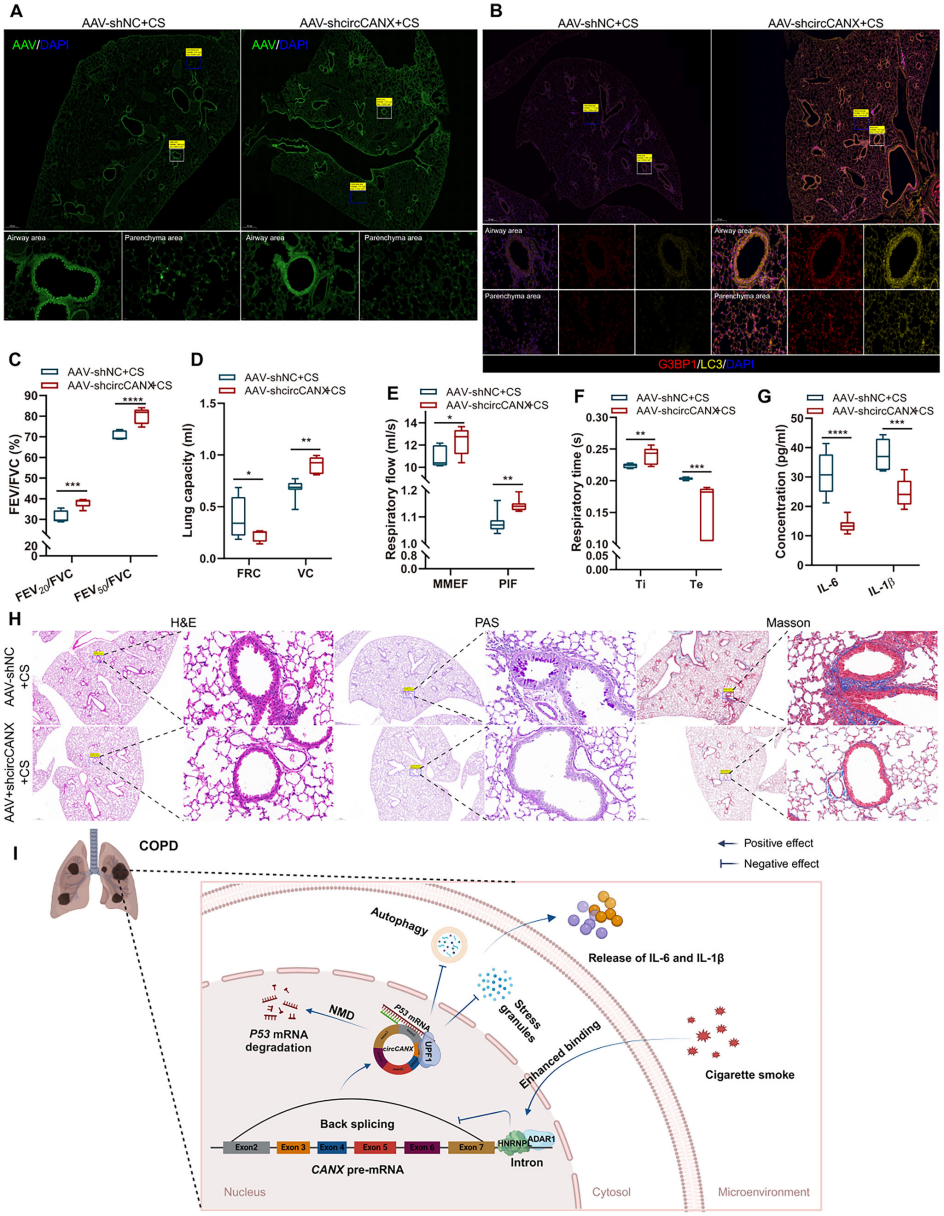
CircCANX inhibition improves destruction of lung structure and inflammation in a mouse model of CS exposure. A) Representative immunofluorescence images of the AAV (FITC‐labeled) infection efficiency of mouse lung tissues. B) Representative immunofluorescence images of G3BP1 (red) and LC3 (yellow) in mouse lungs in AAV‐shNC+CS and AAV‐shcircCANX+CS groups. DAPI was used to stain cell nuclei. C–F) The indicated lung function indices of the two groups (*n* = 7 mice/group). G) ELISAs of IL‐6 and IL‐1β levels in AAV‐shNC+CS and AAV‐shcircCANX+CS groups. H) Representative images of H&E, PAS, and Masson staining of mouse lungs in the indicated groups. I) Schematic diagram: ADAR1‐HNRNPL interaction‐repressed circCANX recruits *P53* mRNA and UPF1, and interacts with each other to assemble a complex in nuclear. This complex promotes *P53* mRNA degradation through the NMD pathway and affects autophagy and SG formation to further regulate inflammation. The schematic diagrams were elaborated by BioRender. Data are presented as mean ± SD values. *p < 0.05, **p < 0.01, ***p < 0.001, ****p < 0.0001.

Next, we constructed a mouse model of CS exposure with circCANX overexpression or circCANX inhibition and its controls using FITC‐labeled AAV‐circCANX and AAV‐vector or FITC‐labeled AAV‐shcircCANX and AAV‐shNC, respectively, and confirmed the effect of virus infection (**Figures** **9**A and **10**A). Lung function analysis showed circCANX overexpression reduced the values of FEV_20_/FVC, FEV_50_/FVC, VC, MMEF, PIF, and Ti, and increased the values of FRC and Te (Figure 9B–E), while circCANX inhibition had the opposite effects on these indexes (Figure 10C–F). Higher counts of neutrophils, macrophages, and lymphocytes were detected in the AAV‐circCANX+CS group than in the AAV‐vector+CS group (Figure 9F). In addition, the weights of mice in the AAV‐circCANX+CS group decreased in the last month (Figure 9G). ELISA assay results demonstrated that the IL‐6 and IL‐1β levels were increased in the CS group, which were augmented in the AAV‐circCANX+CS group (Figure 9H). In contrast, the levels of IL‐6 and IL‐1β were decreased in AAV‐shcircCANX+CS group (Figure 10G). Moreover, circCANX upregulation deteriorated the lung structure of mice with lung emphysematous changes and airway wall thickening, and increased inflammatory cell infiltration, goblet cell proliferation, and lung fibrosis (Figure 9I), while the opposite results were observed after circCANX downregulation (Figure 10H). Furthermore, CS exposure induced LC3 and G3BP1 expression, which was restored by circCANX overexpression (Figure 9J). The LC3 and G3BP1 expression were increased in AAV‐shcircCANX+CS group compared with AAV‐shNC+CS group (Figure 10B). Taken together, these animal experiments suggested that circCANX facilitates the destruction of lung structure and inflammation, and the therapeutic effect of circCANX inhibition in vivo.

## 3 Discussion

COPD is a progressive disease, which inflammation plays important role in its progression.^[^ ^41^, ^42^ ^]^ Inhaled particulate matter, particularly CS, is a major cause of COPD.^[^ ^3^ ^]^ In our study, we successfully constructed a mouse model of CS exposure with exposure to CS for 6 months to simulate CS‐induced COPD disease.^[^ ^43^ ^]^ Autophagy is an inducible response to various stresses that can occur in COPD, such as hypoxia, and particle and CS exposure.^[^ ^10^, ^44^ ^]^ In general, autophagy provides protection to maintain cell homeostasis.^[^ ^10^, ^13^ ^]^ However, during severe COPD, the occurrence of autophagy may represent either a continued adaptive response to cellular stress or an insufficient defense against the pathological process.^[^ ^10^ ^]^ In this study, we first performed functional enrichment analysis, which showed high enrichment for autophagy, and then found that autophagy was increased in CSE‐treated cells and COPD model mice. Rapamycin‐mediated promotion of autophagy inhibited the release of inflammatory factors caused by circCANX, whereas 3‐MA had the opposite effect, indicating that autophagy could relieve circCANX‐mediated inflammation.

CircRNA‐based research has advanced greatly since its inception.^[^ ^45^ ^]^ CircRNAs function in various mechanisms to regulate the development of COPD.^[^ ^46^, ^47^ ^]^ Our study identified a circRNA, circCANX, that was highly stable and downregulated in the peripheral blood of patients with COPD and CES‐treated cells, and its expression was correlated with the FEV_1_/FVE% ratio in patients with COPD. These findings suggested a functional role for this circRNA in disease progression. We also showed that circCANX repressed autophagy and SG formation to regulate inflammation, both in vivo and in vitro.

The human body are equipped with various potent defense mechanisms to maintain host homeostasis and resist damage, for example by reducing harmful gene expression.^[^ ^48^, ^49^ ^]^ In our study, circCANX is a promoter of inflammation, however our results show its expression was decreased in COPD. Therefore, we speculated what might cause this reduction in circCANX expression. CircRNA biogenesis cannot be simply explained by correlating with their linear forms expression.^[^ ^50^ ^]^ In general, the exons generated circRNAs are usually not alternatively spliced.^[^ ^28^, ^51^ ^]^ Thus, circRNA biogenesis competes with canonical mRNA isoforms.^[^ ^23^ ^]^ CircCANX was generated from *CANX* pre‐mRNA and downregulated in COPD, while *CANX* mRNA was upregulated. Thus, we hypothesized that the RNA competition event may result in the decrease of circCANX in COPD. Our data supported that the 2% CSE enhanced ADAR1‐HNRNPL interaction accelerated the canonical linear splicing to generate *CANX* mRNA, which competes with circCANX biogenesis, in this way leading to the decline of circCANX. In addition, autophagy promotes the degradation of misfolded proteins to maintain homeostasis.^[^ ^49^ ^]^ Our results also showed the activation of autophagy partly reduced the circCANX levels. This also contributes to the decline of circCANX in COPD.

Functional circRNAs likely exhibit unique subcellular localizations for various functions.^[^ ^24^ ^]^ For example, nuclear circRNAs participate in transcription and splicing.^[^ ^52^ ^]^ Understanding their subcellular localization patterns in the cytoplasm can also help to predict their functions.^[^ ^53^, ^54^ ^]^ Similarly, P53 has many functions, which rely on its posttranslational modifications and cytosolic and nuclear distribution.^[^ ^55^ ^]^ Posttranslational modifications of P53, such as phosphorylation, ubiquitination, acetylation, and methylation, represent an efficient way on the regulation of apoptosis, ferroptosis, or senescence.^[^ ^55^ ^]^ In addition, nuclear‐located P53 can induce autophagy, while P53 accumulated in the cytoplasm can inhibit autophagy.^[^ ^16^ ^]^ In this study, circCANX was observably localized in the nucleus and combined with the *P53* transcript to promote mRNA destabilization. Notably, this study also indicated that nuclear P53 influenced circCANX‐mediated autophagy, SG formation, and inflammation.

The mRNA surveillance mechanism is imperative for cell growth and function.^[^ ^38^ ^]^ NMD is a characterized and conserved mRNA quality control pathway.^[^ ^56^, ^57^ ^]^ mRNAs containing NMD‐activating features, such as interactions with UPF1, are selected for degradation by NMD.^[^ ^57^, ^58^ ^]^ In our study, circCANX, *P53* mRNA, and UPF1 interacted with each other to promote *P53* mRNA decay and that an NMD‐specific inhibitor or UPF1 deletion could suspend the degradation. This indicates that NMD mediates the circCANX‐induced degradation of *P53* transcript. NMD prefers the regulation of mRNAs with a long 3′‐UTR (>1 kb).^[^ ^57^ ^]^ The major *P53* transcript has a 3′‐UTR of >1.1 kb, which may explain its sensitivity to NMD‐related degradation. In addition, the NMD‐activating factor UPF1 combined with *P53* mRNA can also result in mRNA decay via the NMD pathway.

In this study, circCANX functioned as a scaffold for assembling *P53* mRNA and UPF1 into a ternary complex, leading to *P53* transcript decay. Whether such a complex is specific to the *P53* transcript should be considered. First, according to the immunofluorescence experiments, circCANX, the *P53* transcript, and UPF1 were perfectly co‐localized in the nucleus. Second, circCANX‐induced *P53* degradation was NMD‐dependent, and UPF1 is the key factor in the NMD process.^[^ ^20^ ^]^ Third, circCANX, *P53* mRNA, and UPF1 showed a strong triangular relationship. Based on these firm evidences, we believe that circCANX recruits UPF1 to specific regulate *P53* here. This also reveals the potential of the development of drugs and biomarkers for the clinical management of COPD by focusing on the circCANX‐meidated complex.

In summary, the present study revealed that functional circCANX suppressed P53 expression, and exerted a regulatory effect on inflammation by suspending autophagy and SG formation in vivo and in vitro. Mechanistically, circCANX recruited *P53* mRNA and UPF1, which then interacted to assemble a ternary complex in the nucleus. The circCANX‐*P53* mRNA‐UPF1 complex accelerated *P53* transcript degradation in an NMD‐dependent manner and regulated autophagy, SG formation, and inflammation. Interestingly, linear splicing of *CANX* pre‐mRNA, enhanced by the ADAR1‐HNRNPL interaction, was responsible for the decrease in circCANX biogenesis. Our study provides a new circRNA‐induced mechanism of inflammation regulation in COPD that links mRNA decay, autophagy, SG formation, and circRNA biogenesis. circCANX exits great potential for therapeutic applications as a promoter of inflammation, and its mediated complex and novel regulatory axis offers valuable insights into the progression of COPD. The study further increases our understanding of the therapeutic potency of circRNA and delivers confidence in circRNA‐related drugs and biomarker development in COPD.

## 4 Experimental Section

### Human Blood Samples

Blood samples from patients with COPD (*n* = 52) and healthy controls (*n* = 56) were collected at the First Affiliated Hospital of Anhui Medical University. The diagnosis of COPD followed the guidelines of the Global Initiative for Chronic Obstructive Lung Disease. This study was approved by the Ethics Committee of First Affiliated Hospital of Anhui Medical University (PJ 2024‐07‐56). All participants have signed an informed consent form. Table S1 (Supporting Information) shows the clinical information of the patients.

### RNA‐Sequencing

For circRNA sequencing, blood samples of patients with COPD and healthy controls were immediately mixed with TRIzol Reagent (Invitrogen, 15596026) in a new RNase‐free tube. The RNA library was analyzed by the Illumina HiSeq platform. For transcriptome sequencing, total RNA was isolated from the circCANX overexpression plasmid‐ and control‐treated cells using TRIzol Reagent (Invitrogen, 15596026). The Illumina NovaSeq platform was used for double‐end sequencing in the PE150 mode according to standard protocols.

### Cigarette Smoke Extract (CSE) Preparation

Smoke from Huangshan cigarettes (Tobacco Industrial Corporation, China) was bubbled into a flask containing RPMI‐1640 medium (15 mL) using a vacuum pump with steady speed. The CSE solution was sterilized by 0.22 µm pore filter (Millipore, SVGLA25NB6). Quality control at an absorbance of 540 nm and less than 320 nm, which was between 0.9 OD and 1.2 OD, indicated that the solution was 100% CSE.

### Cell Culture, Isolation, and Transfection

Human bronchial epithelial cells (HBECs) and normal human lung epithelial cells (BEAS‐2B) were purchased from the American Type Culture Collection and cultured in RPMI‐1640 (Gibco, C11875500BT) supplemented with 10% fetal bovine serum (Gibco, 10270‐106). Human pulmonary alveolar epithelial cells (HPAEpiCs) were purchased from OTWO Biotech and cultured in Dulbecco's modified Eagle's medium (Gibco, 11965092) with 10% fetal bovine serum.

Diseased human bronchial epithelial cells (DHBECs) were isolated from the bronchial tissue of patients with COPD. Briefly, bronchial tissues from patients with COPD were obtained and soaked in 1% protease type XIV and 0.01% DNase I solution with rotation at 4 °C overnight. Collagen type IV (0.25 mg mL^−1^), EDTA (2 mM), DTT (0.05 mg mL^−1^), and DNase (10 µg mL^−1^) were added in bronchial tissues to obtain cells. Then, cells were seeded on membranes supported by collagen‐coated 12 mm transwell chamber (Corning Life Sciences, USA) after culturing in dishes coated with type I rat‐tail collagen. Finally, the bronchial epithelial cells were identified by immunofluorescence with epithelial cell‐specific protein of CK17/19. All cells were cultured in a humidified incubator containing 5% CO2 at 37 °C.

siRNAs and their controls (GenePharma, Shanghai, China) were transfected by Lipofectamine RNAiMAX reagent (Invitrogen Life Technologies, 13778075). sgRNAs (Tsingke Biotech, China), overexpression plasmids (GenePharma), and the corresponding negative controls were transfected with Nano Trans 40 (Biomedical, B1002). Table S2 (Supporting Information) lists the information of all constructs used in this study. Adenovirus‐mCherry‐GFP‐LC3 (Beyotime Biotechnology, C3011) were infected in cells (MOI: 15) to monitor autophagy flux. The signals were imaged by laser confocal microscope (LSM980, Carl Zeiss, Oberkochen, Germany).

### Cells Treatment and Activator/Inhibitor Incubation Assays

For CSE treatment, cells were exposed to CSE at concentrations of 1%, 2%, 3%, and 4% for 24 h. For RNase R treatment, total RNA was incubated at 37 °C for 15 min with 2 U µg^−1^ RNase R (Beyotime Biotechnology, R7092S). For actinomycin D treatment, cells were cultured with 2 µg mL^−1^ actinomycin D (MedChemExpress, HY‐17559) for specific times. For the activator/inhibitor incubation assays, all activators and inhibitors were prepared per the manufacturer's instructions. Cells were treated with the autophagy activator rapamycin (20 nm; MedChemExpress, HY‐10219) or inhibitor 3‐MA (10 mm; MedChemExpress, HY‐19312) for 24 h. Cells were treated with the NMD inhibitor NMDI14 (5 or 10 µm; MedChemExpress, HY‐111374) for 48 h.

### RNA Extraction and Quantitative Real‐Time PCR (RT‐qPCR)

Total RNA was extracted from the cells by TRIzol reagent (Invitrogen, 15596026), and then synthesized to cDNA by Hifair III 1st Strand cDNA Synthesis SuperMix (Yeasen Biotechnology, HB210716). RT‐qPCR assays were performed on the Light Cycler 480 (Roche Applied Science). *U6* and *Actb/β‐actin* were regarded as a reference gene for normalizing data. The primers (Sangon Biotech, Shanghai, China) used in this study are listed in Table S2 (Supporting Information).

### Cell Counting Kit‐8 (CCK‐8) Assay

CSE‐treated cells were seeded into 96‐well plates at a density of 1500 cells in medium (100 µL per well) for 24 h. CCK‐8 reagent (Beyotime Biotechnology, C0042) was incubated with cells at 37 °C. Cell viability was assessed at absorbance at 450 nm by microplate reader (Bio‐Tek Elx800, USA).

### Fluorescence In Situ Hybridization (FISH) and Immunofluorescence Staining

Cells seeded in confocal dish (NEST, 801002) were fixed with 4% paraformaldehyde (Beyotime Biotechnology, P0099) and permeabilized by 0.5% Triton X‐100 (Beyotime Biotechnology, P0096) for 10 min. For FISH, cells were incubated with a FITC‐labeled circCANX probe (GenePharma, F12202) or Cy3‐labeled *P53* mRNA probe (Tsingke Biotech, NJP23122000004) at 37 °C overnight. For immunofluorescence staining, cells were incubated with primary antibodies at 4 °C overnight. Then the incubated secondary antibodies were added for 1 h at 37 °C. DAPI was added to the anti‐fade mounting medium. The signals were imaged by laser confocal microscope (LSM980, Carl Zeiss, Oberkochen, Germany).

### Western Blot

Cells were lysed in RIPA buffer (Beyotime Biotechnology, P0013B) blending PMSF (Beyotime Biotechnology, ST505) for protein samples preparation. Equal qualities of protein were electrophoresed using 12.5% SDS‐PAGE gels. A PVDF membranes (Millipore, ISEQ00005) was subjected to transfer proteins and then incubated with indicated primary antibodies with rotation at 4 °C overnight. These membranes were then added with the corresponding horseradish peroxidase‐labeled secondary antibodies for 1 h. Finally, protein signals were detected using Omni‐ECL (Epizyme, SQ101). Table S3 (Supporting Information) lists all antibodies used in this study.

### ELISA Assay

The supernatant from the different treatment groups was collected to detect the IL‐6 and IL‐1β levels using a Human IL‐6 High Sensitivity ELISA Kit (Multi Sciences, EK106HS) and Human IL‐1β ELISA Kit (Proteintech, KE00021) according to the manufacturer's instructions. The BALF of mice was used to detect the IL‐6 and IL‐1β levels using a Mouse IL‐6 ELISA kit (Weiao Biotechnology, EM30325 M) and Mouse IL‐1β ELISA Kit (Multi Sciences, EK201B).

### RNA‐Binding Protein Immunoprecipitation (RIP)

RIP assays were performed with the EZ‐Magna RIP RNA‐Binding Protein Immunoprecipitation Kit (Millipore, 17‐701). Treated cells were lysed in RIP lysis buffer blending protease and RNase inhibitors. The beads conjugated with IgG or primary antibodies were added to the cell lysates with rotation at 4 °C for 12 h. Then, the immunoprecipitate was washed by wash buffer and mixed in proteinase K. Finally, RNA was extracted using the Trizol Reagent (Invitrogen, 15596018).

### RNA Pull‐Down and Purification

Cell lysates, prepared using IP lysis buffer, were subjected to RNA pull‐down using a Pierce Magnetic RNA‐Protein Pull‐Down Kit (Thermo Fisher Scientific, 20164). After the Pierce Streptavidin Magnetic Beads were incubated with probes for 30 min, then the beads were added to the cell lysates with rotation at 4 °C for 12 h. For mass spectrometry (MS) assays, the RNA‐bead complexes were immobilized on 10% SDS‐PAGE gels. For the western blot analysis, the RNA‐bead complexes were boiled with loading buffer for sample preparation. For agarose gel electrophoresis, TRIzol reagent (Invitrogen, 15596026) was subjected to extract RNA from the beads.

### Mass Spectrometry (MS) Analysis

Gel pieces were dehydrated with acetonitrile and reduced with dithiothreitol. Following alkylation with iodoacetamide, gel pieces were soaked in trypsin to extract peptides. The peptide samples were analyzed on an Orbitrap Eclipse (Thermo Fisher Scientific) by QLBio Co., Ltd. The analyzer was operated with the following settings: a resolution of 120000 at a mass/charge ratio of 200 was set for MS1 and 1T for MS2, the automatic gain control target was set to 1.0 × 10^+6^ with maximum inject time of 20 ms. The most intense ions were fragmented by HCD with an isolation window of 0.4 m/z and were excluded for 30 s. Acquired raw data were analyzed using MaxQuant software version (v1.6.2.10).

### Immunoprecipitation (IP)

Treated cells were lysed using RIPA buffer (Beyotime Biotechnology, P0013B). The cell lysates incubated with normal IgG and the indicated primary antibodies with rotation at 4 °C overnight. The antibody–protein immunoprecipitate was generated by protein A + G beads (Beyotime Biotechnology, P2108) for 2 h before western blotting.

### Adeno‐Associated Virus (AAV) Construction

FITC‐labeled AAV‐circCANX and AAV‐shcircCANX and their controls were constructed by Genomeditech (Shanghai, China). The sequence and RNA interference sequence of circCANX were inserted into the rAAV expression plasmid to obtain target plasmids. The target AAV plasmids were then transfected into AAV Pro‐293T cells with a serum type plasmid, an adenovirus helper plasmid, and HG transgene reagent mixture. After transfection for 6–8 h, the enhanced buffer was added to the cells and culture continued for 72 h. Finally, the virus‐rich cells and supernatant were concentrated and purified to obtain high titer virus (3.54 × 10^+12^ VG mL^−1^). For animal experiments, mice were intratracheally instilled with 1 × 10^+11^ VG per mouse in the last month of CS treatment. The effect of virus infection was confirmed by observing green fluorescence in mouse lung sections.

### Animal Experiments

Eight‐week‐old male C57BL/6 mice were randomly divided into six groups: control (Con), CS, AAV‐vector+CS, AAV‐circCANX+CS, AAV‐shNC+CS, and AAV‐shcircCANX+CS (*n* = 7 mice/group). Mice in the last five groups were exposed to CS (300 mg m^−3^ of total particulate matter) in a whole‐body exposure system for 90 min twice a day, 4 h apart, 6 days a week for 6 months. Air‐exposed mice served as nonsmoking controls. All mice were bred in the Animal Experiment Center of Anhui Medical University, under a 12 h light/dark cycle with free access to water and food. For AAV‐circCANX+CS, AAV‐shcircCANX+CS and its controls groups, sterile saline (containing 1 × 10^+11^ virus particles) was instilled intratracheally to each mouse in the last month of treatment. After 6 months, mice were euthanized with sodium pentobarbital (50 mg kg^−1^). A blunt‐tipped catheter was inserted into the trachea through which 4% polyformaldehyde was instilled into the lung. The whole lungs were then removed and placed on ice before processing. The animal experiments were approved by the Animal Ethics Committee of Anhui Medical University (LLSC20242225).

### Histological Staining and Immunohistochemistry (IHC)

Lung tissues from mice were cut for slides. These slides were added with xylene for deparaffinization and graded ethanol for rehydration. For the histological staining, hematoxylin and eosin (H&E) staining was performed with H&E staining kit (Beyotime Biotechnology, C0105S) following the manufacturer's instructions. Periodic acid‐Schiff (PAS) staining was performed using the PAS staining kit (Beyotime Biotechnology, C0142S), and Masson staining was accomplished using the Trichrome staining kit (Beyotime Biotechnology, C0189S). For IHC staining, the slides were blocked endogenous peroxidase activity with 3% hydrogen peroxide. After soaking in sodium citrate buffer under high pressure for antigen recovery, the slides treated with 5% bovine serum albumin. The sections were then incubated with anti‐P53 primary antibody at 4 °C overnight, followed by incubation with the secondary antibody. Images were captured using a VS200 microscope (Olympus, Tokyo, Japan).

### Bioinformatics Analysis

CircCANX sequence data were analyzed by circBase (http://www.circbase.org/). For the circRNA‐mRNA interaction simulation and analysis, Mfold (version 2.3, http://unafold.rna.albany.edu/?q=mfold) was used to calculate the secondary structures with minimum free energy. The best secondary structure for circCANX would have a free energy of −176.10 kcal mol^−1^. RNA Composer (http://rnacomposer.cs.put.poznan.pl/) was used to generate a 3D model. For circRNA‐protein interaction simulation and analysis, a 3D model of circCANX and UPF1 (PDB No. 2GJK) was generated using HDOCK software (http://hdock.phys.hust.edu.cn/). The two atoms were considered to interact if the distance between them was <4.5 Å.

### Statistical Analysis

Experiments were repeated with at least three biological replicates. GraphPad Prism 8.0 and SPSS 23.0 were conducted for statistical analysis. Differences between two groups were analyzed using Student's *t*‐test, while differences between multiple groups were estimated using one‐way or multi‐factor analysis of variance (ANOVA). Correlations between circCANX and *P53* mRNA expression were analyzed using Pearson's correlation test. Data are presented as mean ± SD values. Differences were considered statistically significant at *p < 0.05, **p < 0.01, ***p < 0.001, ****p < 0.0001.

## Conflict of Interest

The authors declare no conflict of interest.

## Author Contributions

T.‐T.C., Y.‐Y.W., and J.‐Y.K. contributed equally as joint first authors. T.‐T.C., Y.‐Y.W., and J.‐Y.K. participated in the experiments, analyzed data, and drafted the manuscript. D.‐W.Z., J.‐J.Y., and X.‐S.S. provided constructive comments and discussion. M.H. and W.‐T.Z. contributed to the sample collection. G.‐H.F., Z.‐X.D., and H.‐M.W. conceived and designed the experiments and revised the manuscript.

## Supporting information



Supporting Information

## Data Availability

The data that support the findings of this study are available from the corresponding author upon reasonable request.
