# Supplementary material for: ADAR1‐HNRNPL‐Mediated CircCANX Decline Promotes Autophagy in Chronic Obstructive Pulmonary Disease
Source: Adv Sci (Weinh). 2025 Mar 17;12(18):2414211. doi: 10.1002/advs.202414211 (PMC12079403; doi:10.1002/advs.202414211)
Supplement: Supplementary file 1 — Supporting Information [file ADVS-12-2414211-s001.docx]

**ADAR1-HNRNPL-Mediated CircCANX Decline Promotes Autophagy in Chronic Obstructive Pulmonary Disease**

Ting-Ting Chen^1, 2, #^, Yuan-Yuan Wei^1, 2, #^, Jia-Ying Kang^1, 2, #^, Da-Wei Zhang^1, 2^, Jing-Jing Ye^1, 2^, Xi-Shi Sun^5^, Mei Hong^1, 2^, Wen-Ting Zhang^1, 2^, Hui-Mei Wu^4, *^, Zhen-Xing Ding^3, *^, Guang-He Fei^1, 2, *^

1 Department of Respiratory and Critical Care Medicine, First Affiliated Hospital of Anhui Medical University, Hefei 230022, Anhui Province, China

2 Key Laboratory of Respiratory Diseases Research and Medical Transformation of Anhui Province, Hefei 230022, Anhui Province, China

3 Department of Emergency Medicine, First Affiliated Hospital of Anhui Medical University, Hefei 230022, Anhui Province, China

4 Department of Geriatric Respiratory and Critical Care Medicine, First Affiliated Hospital of Anhui Medical University, Hefei 230022, Anhui Province, China

5 Emergency Medicine Center, Affiliated Hospital of Guangdong Medical University, Zhanjiang 524000, Guangdong Province, China

#These authors contributed equally as joint first authors

***Corresponding author:**

**Guang-He Fei**

Department of Respiratory and Critical Care Medicine, First Affiliated Hospital of Anhui Medical University, Hefei, 230022, Anhui Province, China

Key Laboratory of Respiratory Diseases Research and Medical Transformation of Anhui Province, Hefei, 230022, Anhui Province, China

Email: [gh.fei@ahmu.edu.cn](mailto:gh.fei@ahmu.edu.cn)

**Zhen-Xing Ding**

Department of Emergency Medicine, First Affiliated Hospital of Anhui Medical University, Hefei 230022, Anhui Province, China

Email: dingzhenxing@ahmu.edu.cn

**Hui-Mei Wu**

Department of Geriatric Respiratory and Critical Care Medicine, First Affiliated Hospital of Anhui Medical University, Hefei 230022, Anhui Province, China

Email: [wuhm@ahmu.edu.cn](mailto:wuhm@ahmu.edu.cn)

Supporting Information of Figures


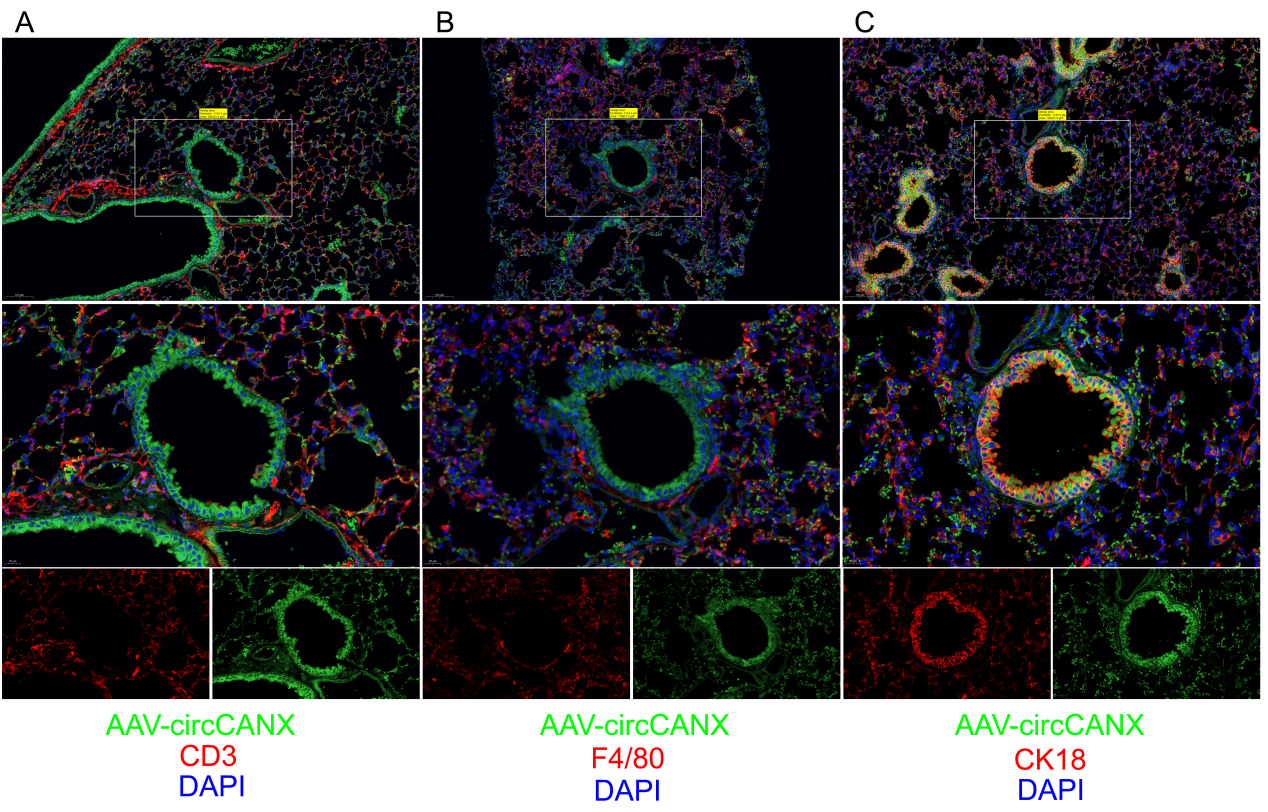


**Figure S1.** CircCANX is co-located with lymphocytes, macrophages, and epithelial cells in the lung of CS exposure model mice. A-C) Immunofluorescence staining of CD3 (red), F4/80 (red), and CK18 (red) and co-located with FITC-labeled AAV-circCANX in the lung tissue of CS-exposed mice. DAPI was used to stain cell nuclei.


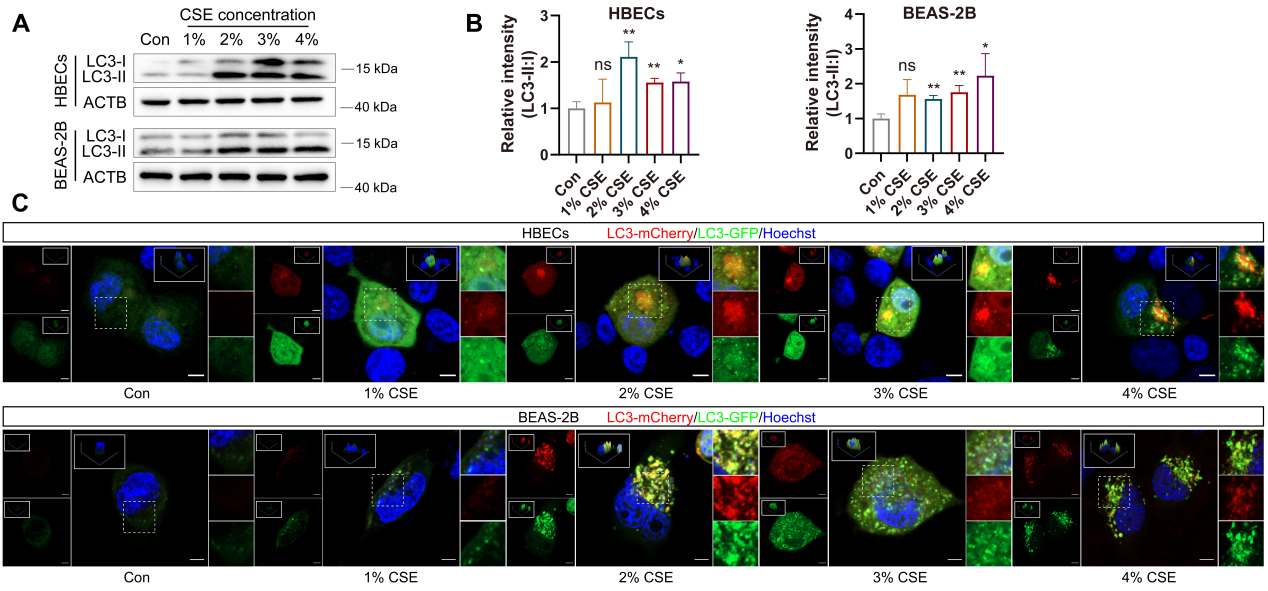


**Figure S2.** The effect of CSE concentration on autophagy. A-B) Western blot analysis and quantitation of autophagy-related protein LC3 in cells with CSE treated at several concentration. C) LC3 dots in mCherry-GFP-LC3-transfected cells in the indicated groups. Hoechst dye was used to stain cell nuclei. Scale bar: 5 µm. Data are presented as mean ± SD values. **p* < 0.05, ***p* < 0.01.

**Figure S3.** circCANX suppresses autophagy and increases the levels of IL-6 and IL-1β in cells. A) Western blot analysis and quantitation of LC3 protein in DHBECs cells after circCANX overexpression and knockdown. B) LC3 dots in mCherry-GFP-LC3-labeled DHBECs cells in the indicated groups. Hoechst dye was used to stain cell nuclei. Scale bar: 5 µm. C) ELISAs of IL-6 and IL-1β levels in DHBECs with indicated treatments. D-E) RT-qPCR detection of *IL-6* and *IL-1β* mRNA in cells in the indicated groups. Data are presented as mean ± SD values. **p* < 0.05, ***p* < 0.01, ****p* < 0.001, *****p* < 0.0001.


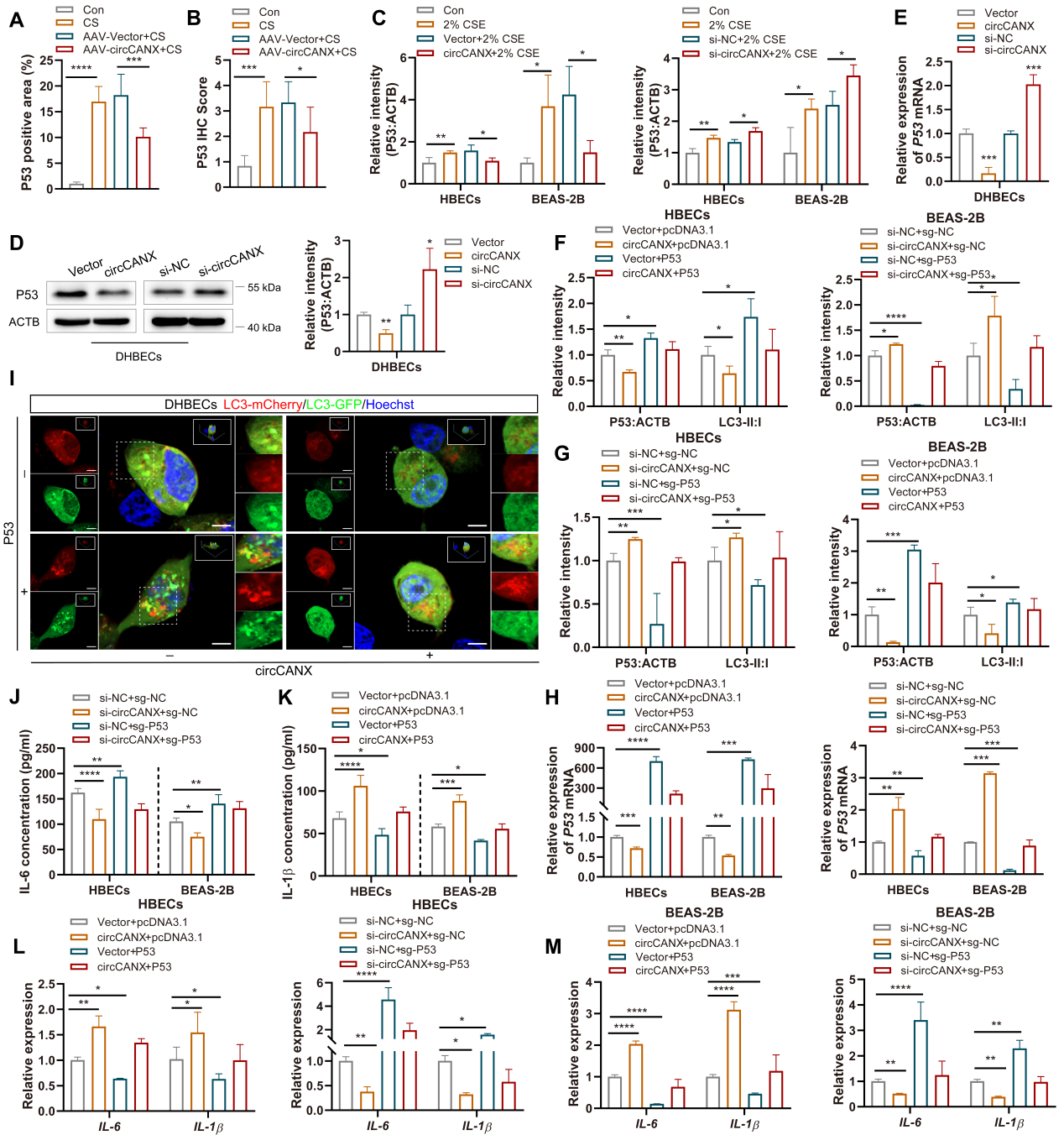


**Figure S4.** P53 is suppressed by circCANX and functionally relevant. A-B) Quantitative analysis and IHC score of P53 protein in immunohistochemistry experiment. C) Quantitative analysis of P53 protein in normal and 2% CSE-treated cells transfected with circCANX plasmid, siRNA, and its controls. D-E) Expression of P53 at both the protein and mRNA levels was detected by western blotting and RT-qPCR in DHBECs. F-G) Quantitative analysis of P53 and LC3 proteins in treated cells. H) RT-qPCR detection of *P53* mRNA in cells from the indicated groups. I) LC3 dots after mCherry-GFP-LC3 transfection in DHBECs. Hoechst dye was used to stain cell nuclei. Scale bar: 5 µm. J-K) ELISAs of IL-6 (J) and IL-1β (K) expression in cells. L-M) Relative expression of *IL-6* and *IL-1β* mRNA were detected by RT-qPCR in the indicated groups. Data are presented as mean ± SD values. **p* < 0.05, ***p* < 0.01, ****p* < 0.001, *****p* < 0.0001.


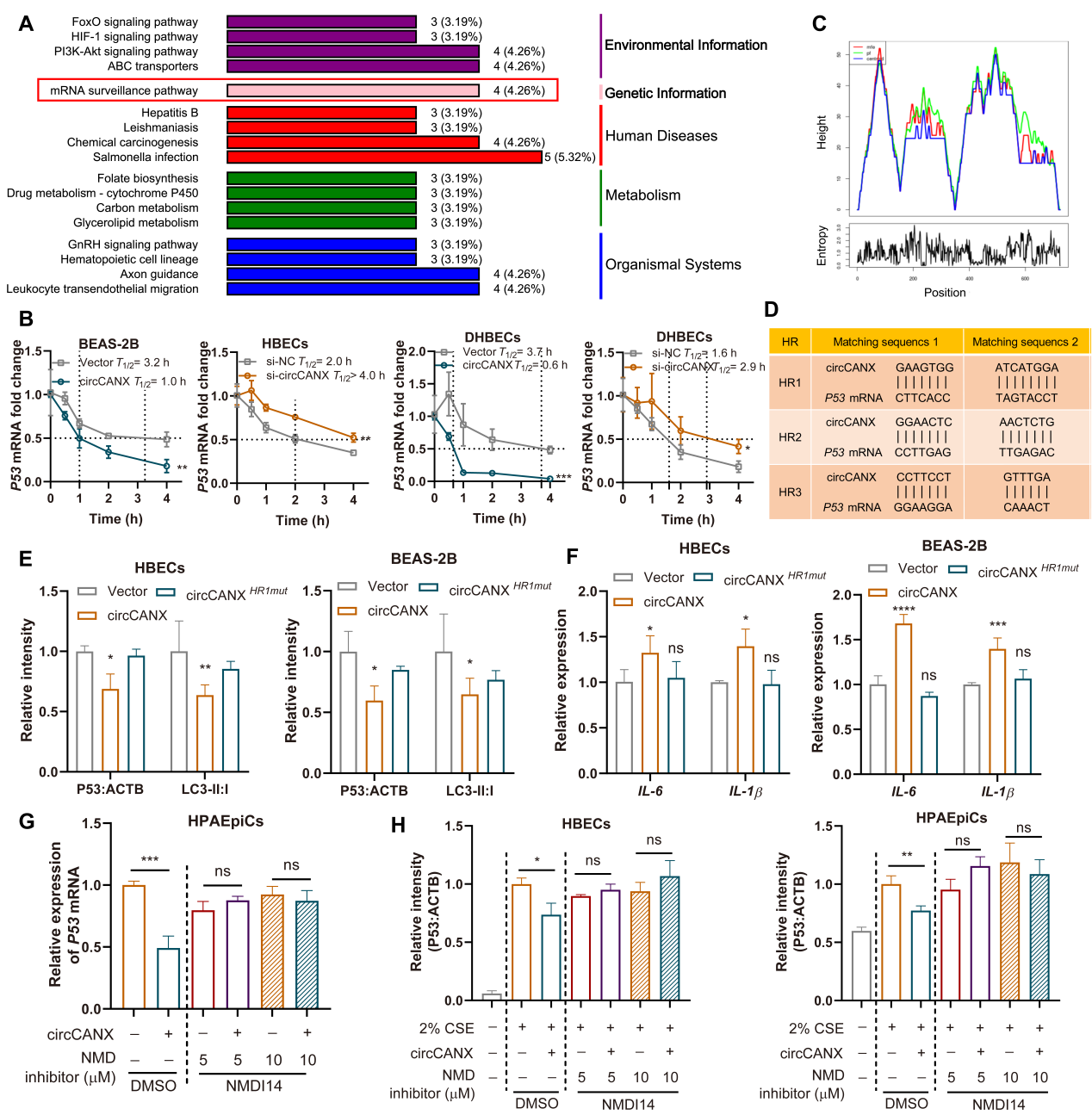


**Figure S5.** CircCANX promotes mRNA decay by physically connecting the *P53* transcript in the NMD-related manner. A) Pathway enrichment analysis of circCANX-interacting genes detected by transcriptome analysis. B) RT-qPCR detection of *P53* mRNA in circCANX silence and overexpression cells with treated with actinomycin D for the indicated times. The dashed line showed the half-life of *P53* mRNA. C) Mountain plot showing the MFE structure (upper) and the entropy (lower) for each position in circCANX. D) Diagram showing HRs-*P53* mRNA matching sequences. E) Quantitative analysis of P53 and LC3 proteins in cells in the Vector, circCANX, and circCANX*^HR1mut^* groups. F) RT-qPCR detection of *IL-6* and *IL-1β* mRNA in the indicated groups. G) Relative expression of *P53* mRNA in HPAEpiCs cells co-treated circCANX plasmid or its control with NMDI14. H) Quantitative analysis of P53 proteins in cells with indicated treatment. Data are presented as mean ± SD values. **p* < 0.05, ***p* < 0.01, ****p* < 0.001, *****p* < 0.0001.


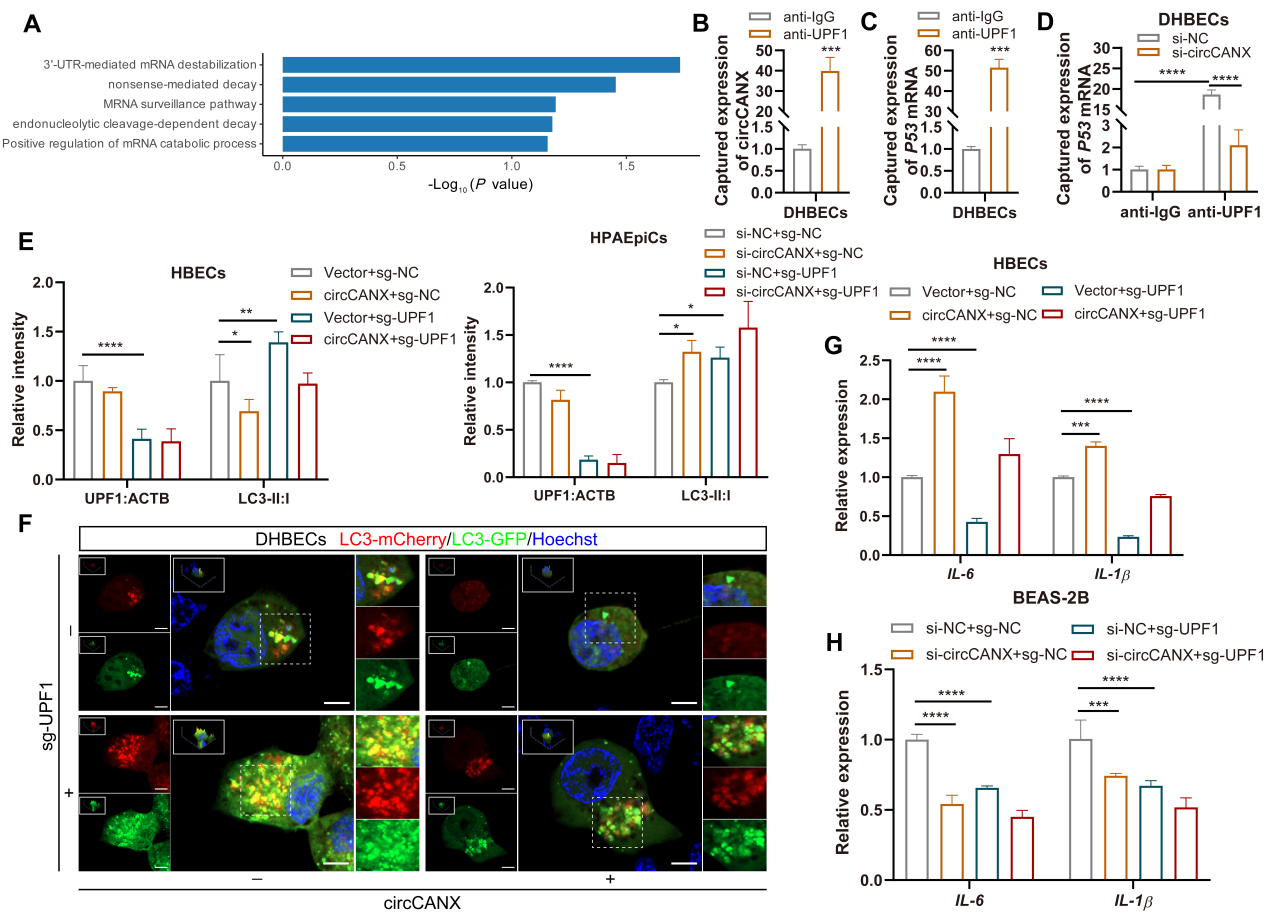


**Figure S6.** UPF1 stabilizes the circCANX-*P53* mRNA interaction by forming a ternary complex. A) Pathway and functional enrichment analysis of UPF1, as determined by MS. B) RIP assay of circCANX expression after UPF1 immunoprecipitation in DHBECs. C-D) RIP assay of *P53* mRNA expression after using the anti-UPF1 with or without circCANX konckdown in DHBECs. E) Quantitative analysis of UPF1 and LC3 proteins in UPF1 knockout cells with circCANX silence and overexpression treatment. F) LC3 dots in mCherry-GFP-LC3-transfected DHBECs cells in the indicated groups. Hoechst dye was used to stain cell nuclei. Scale bar: 5 µm. G-H) Relative expression of *IL-6* and *IL-1β* mRNA were detected by RT-qPCR in the indicated groups. Data are presented as mean ± SD values. **p* < 0.05, ***p* < 0.01, ****p* < 0.001, *****p* < 0.0001.


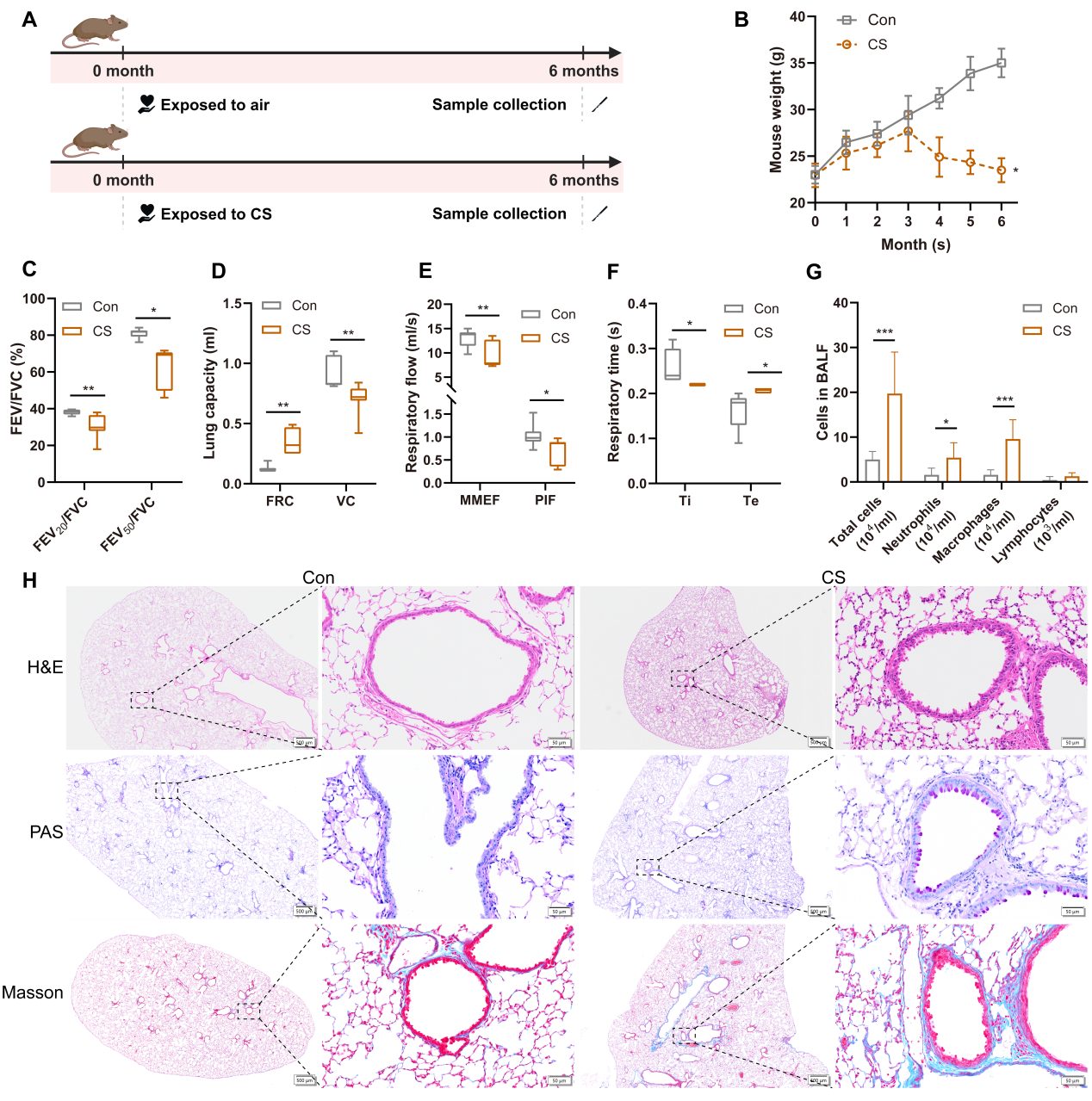


**Figure S7.** The construction of a mouse model of CS exposure. A) Schematic workflow of CS (or air) exposure treatment in C57BL/6 mice for 6 months (*n* = 7 mice/group) to construct mouse model of CS exposure. B) Mouse weight recorded monthly in the two groups. C-F) Lung function indices, including FEV_20_/FVC and FEV_50_/FVC (C), FRC and VC (D), MMEF and PIF (E), and Ti and Te (F) in control and CS-treated groups. G) The cell classification and counting in BALF in the two groups. H) Representative images of H&E, PAS, and Masson staining of mouse lungs in control and CS groups. Data are presented as mean ± SD values. **p* < 0.05, ***p* < 0.01, ****p* < 0.001.

Supporting Information of Tables

**Table S1.** Clinical information for blood donors.

| Clinical information | Con | COPD |
| --- | --- | --- |
| All cases | 56 | 52 |
| Gender |  |  |
| Male | 47 | 41 |
| Female | 9 | 11 |
| Age (year) | 62.93 ± 11.39 | 71.87 ± 8.787*** |
| BMI | 22.81 ± 3.511 | 21.18 ± 3.559 |
| Smoking status | 8.929 ± 10.82 | 17.31 ± 14.70**** |
| FEV1/FVC (%) | 82.24 ± 6.784 | 51.13 ± 11.02^**^ |
| FEV1 (%) predicted | 91.48 ± 11.80 | 38.68 ± 15.96**** |
| GOLD status |  |  |
| I | / | 1 |
| II | / | 9 |
| III | / | 22 |
| IV | / | 20 |

Data are shown as the means ± SD. ***p* < 0.01, ****p* < 0.001, *****p* < 0.0001.

| **Table S2.** Primers, oligo, probes, and plasmids used in our experiments. | |
| --- | --- |
| Gene | Sequence (5’-3’) or supplier |
| *β-actin* | F: AGCGAGCATCCCCCAAAGTT |
|  | R: GGGCACGAAGGCTCATCATT |
| *U6* | F: CTCGCTTCGGCAGCACA |
| *circCANX* | F: ACACACTAAATCATGGAAGGGA |
|  | R: GATGAAGGAGGAGCAGTGGT |
| linear *CANX* | F: GTAGCCCTTCCTGTGTTCCT |
|  | R: TGACAGTGCCACCATCTTCT |
| *CANX* pre-mRNA | F: CTTGGCCATCTGTTGATCCG |
|  | R: TTGTCTTCAACCCCTCTCCC |
| *P53* | F: GTTCCGAGAGCTGAATGAGG |
|  | R: TCTGAGTCAGGCCCTTCTGT |
| *ADAR1* | F: AGCCAAAGACACTCCCTCTC |
|  | R: ATTCCCTGTTCCCAAGCTGA |
| *IL-6* | F: AGTCCTGATCCAGTTCCTGC |
|  | R: CTACATTTGCCGAAGAGCCC |
| *IL-1β* | F: CAGAAGTACCTGAGCTCGCC |
|  | R: AGATTCGTAGCTGGATGCCG |
| *CYP1B1* | F: CTCTCTGCGGAAAAGAAGGC |
|  | R: CCTGATCCAATTCTGCCTGC |
| *HMOX1* | F: ATGCCCCAGGATTTGTCAGA |
|  | R: AAGTAGACAGGGGCGAAGAC |
| *SNX22* | F: TTCCTGAGACTTCGGCACTT |
|  | R: TGGGCTTTATCTGGGCTGAT |
| *CXCL8* | F: CAGTTTTGCCAAGGAGTGCT |
|  | R: ACTTCTCCACAACCCTCTGC |
| *BMP6* | F: AGAAGAAGGCTGGCTGGAAT |
|  | R: GAAGGGCTGCTTGTCGTAAG |
| *PPP2R3B* | F: GAGCAGATGGACGAACTTGG |
|  | R: AAGCGCCCCACATTGATAAC |
| *PTPN22* | F: TTGAACCAGGAGTCAGCTGT |
|  | R: CTTTCTGGGAGAGGAGGTGG |
| *AMT* | F: GGGTGAAGCTGATGGAGAGT |
|  | R: GTTCTGAAGCTCCCTGACCT |
| *CRELD1* | F: GCATCTGTGTGAAGGAGCAG |
|  | R: AGAAGAAGCCTGCTGACTGT |
| *Negative control* | sense: UUCUCCGAACGUGUCACGUTT |
|  | antisense: ACGUGACACGUUCGGAGAATT |
| *si-circCANX* | sense: UCUUUACACACUAAAUCAUTT |
|  | antisense: AUGAUUUAGUGUGUAAAGATT |
| *si-ADAR1* | sense: AGCCAAAGACACTCCCTCTCTT |
|  | antisense: ATTCCCTGTTCCCAAGCTGATT |
| *circCANX* fish probe with FITC conjugated | T+TCCATGAT+TTAGTGTGTAAAGATG |
| *circCANX HR1* probe | ATCATGGAAGGGAAGTGGTTGCTGT |
| *circCANX HR2* probe | TGACAGAGGAACTCTGTCA |
| *circCANX HR3* probe | AGCCCTTCCTGTTTGACACCAAGCCTCTCAT |
| *P53* mRNA probe with Cy5 conjugated | CAGCCTCTGGCATTCTGGGAGCTTCATCTGGACCTG |
| Plasmid: *circCANX* | Gene Pharma |
| Plasmid: *P53 pcDNA 3.1* | Gene Pharma |
| Plasmid: *ADAR1 pcDNA 3.1* | Gene Pharma |
| Plasmid: *circCANX ^HR1mut^* | TsingKe Biotech |
| *Plasmid: circCANX ^UPF1mut^* | TsingKe Biotech |
| *Plasmid: sg-P53 pSpCas9(BB)-2A-Puro (PX459) V2.0* | TsingKe Biotech |
| *Plasmid: sg-UPF1 pSpCas9(BB)-2A-Puro (PX459) V2.0* | TsingKe Biotech |

**Table S3.** Antibodies used in the present study.

| Product | Source | No. of Catalogue |
| --- | --- | --- |
| Western blot: | | |
| anti-ACTB | HUABIO | EM21002 |
| anti-P53 | Proteintech | 10442-1-AP |
| anti-UPF1 | Proteintech | 23379-1-AP |
| anti-G3BP1 | Proteintech | 66486-1-Ig |
| anti-HNRNPL | Proteintech | 18354-1-AP |
| anti-LC3 | Cell Signaling Technology | 12741S |
| anti-ADAR1 | Cell Signaling Technology | 81284S |
| Secondary antibody | | |
| Anti-rabbit IgG, HRP-linked Antibody | Cell Signaling Technology | 7074P2 |
| Anti-mouse IgG, HRP-linked Antibody | Cell Signaling Technology | 7076P2 |
| Goat Anti-Rabbit lgG AF 594 | Abmart | M21014 |
| Goat Anti-Mouse lgG AF 594 | Abmart | M21013 |
| Goat Anti-Mouse lgG AF 488 | Abmart | M21011 |
